# Effects of Elevated CO_2_ on Nutritional Quality of Vegetables: A Review

**DOI:** 10.3389/fpls.2018.00924

**Published:** 2018-08-15

**Authors:** Jinlong Dong, Nazim Gruda, Shu K. Lam, Xun Li, Zengqiang Duan

**Affiliations:** ^1^State Key Laboratory of Soil and Sustainable Agriculture, Institute of Soil Science, Chinese Academy of Sciences, Nanjing, China; ^2^Division of Horticultural Sciences, Institute of Crop Science and Resource Conservation, University of Bonn, Bonn, Germany; ^3^School of Agriculture and Food, Faculty of Veterinary and Agricultural Sciences, The University of Melbourne, Melbourne, VIC, Australia

**Keywords:** antioxidants, climate change, elevated carbon dioxide, environmental factors, greenhouse vegetables, mineral, protein, soluble sugar

## Abstract

Elevated atmospheric CO_2_ (eCO_2_) enhances the yield of vegetables and could also affect their nutritional quality. We conducted a meta-analysis using 57 articles consisting of 1,015 observations and found that eCO_2_ increased the concentrations of fructose, glucose, total soluble sugar, total antioxidant capacity, total phenols, total flavonoids, ascorbic acid, and calcium in the edible part of vegetables by 14.2%, 13.2%, 17.5%, 59.0%, 8.9%, 45.5%, 9.5%, and 8.2%, respectively, but decreased the concentrations of protein, nitrate, magnesium, iron, and zinc by 9.5%, 18.0%, 9.2%, 16.0%, and 9.4%. The concentrations of titratable acidity, total chlorophyll, carotenoids, lycopene, anthocyanins, phosphorus, potassium, sulfur, copper, and manganese were not affected by eCO_2_. Furthermore, we propose several approaches to improving vegetable quality based on the interaction of eCO_2_ with various factors, including species, cultivars, CO_2_ levels, growth stages, light, O_3_ stress, nutrient, and salinity. Finally, we present a summary of the eCO_2_ impact on the quality of three widely cultivated crops, namely, lettuce, tomato, and potato.

## Introduction

The atmospheric CO_2_ concentration has increased from 280 μmol mol^-1^ before the industrial revolution to 408 μmol mol^-1^ now^1^ (March 2018) mainly due to fossil fuel combustion and deforestation. It is predicted to reach 1000 μmol mol^-1^ by the end of this century (IPCC, 2014). Elevated CO_2_ (eCO_2_) can promote net photosynthetic rates of plants and thus plant productivity and yield (Long et al., 2004). It also enhances plant tolerance to environmental stresses via increased soluble sugars, antioxidants, and root exudates (Drake et al., 2011; Huang and Xu, 2015). Therefore, eCO_2_ has been widely used as a gas fertilizer in greenhouse vegetable cultivation, particularly in recent decades as greenhouse technologies have improved (Mortensen, 1987; Bisbis et al., 2018) and the demand for vegetables is continuously increasing (**Supplementary Figure S1**).

Generally, eCO_2_ (700–1000 μmol mol^-1^) can promote the yield of vegetables (Gruda and Tanny, 2014). The sources of CO_2_ have changed from traditional straw bales and organic soils to relatively pure CO_2_ from industrial waste or CO_2_ generators (Gruda, 2005). Elevated CO_2_ has frequently been demonstrated to increase the yield of various crops, including vegetables (Kimball, 1983; Long et al., 2004). Elevated CO_2_ (from 355 to 800–900 μmol mol^-1^) increased the yield of lettuce, carrot, and parsley by 18%, 19%, and 17%, respectively (Mortensen, 1994). Optimizing other environmental factors with eCO_2_ further increased plant productivity and yield (Kirschbaum, 2011). Elevated CO_2_ (900 μmol mol^-1^) with additional light (ambient + 100 μmol m^-2^ s^-1^ photosynthetically active radiation or PAR) increased the early yield of tomato and pepper by 15% and 11%, respectively (Fierro et al., 1994). Elevated CO_2_ (600–700 μmol mol^-1^) increased the average root dry mass of sugar beet by 26% in high N availability (10 mM NO_3_^-^) and by 12% in 1 mM NO_3_^-^ (Demmers-Derks et al., 1998). More examples of yield benefits for other vegetable crops are reviewed by Gruda (2005).

However, there is less information on the effect of CO_2_ concentration on the nutritional quality of vegetables (Gruda, 2005; Moretti et al., 2010). The effect of eCO_2_ on vegetable quality has been briefly reviewed (Idso and Idso, 2001; Gruda, 2005; Moretti et al., 2010; Bisbis et al., 2018). However, these reviews mainly focus on limited parameters of quality affected by various environmental factors. A comprehensive review of recent studies explaining and targeting the key role of the effect of eCO_2_ on vegetable quality is lacking. To address this knowledge gap, we conducted a meta-analysis on the eCO_2_ effect and its interaction with factors besides eCO_2_ on the quality of vegetables, and more specifically of three widely cultivated vegetables: lettuce, tomato, and potato. This information is critical for vegetable nutrition and food security under future climate change.

## Methodology

### Data Collection

A literature search was conducted for publications between 1990 and 2018 using the following databases: Web of Knowledge, Scopus, ScienceDirect, and Google Scholar. The keywords used were “vegetable,” “vegetable quality,” “quality,” “elevated carbon dioxide,” “eCO_2_,” “CO_2_ enrichment,” “FACE,” and “climate change.” The environmental factors “CO_2_ level,” “CO_2_ concentration,” “light intensity,” “light quality,” “temperature,” “heat stress,” “chilling stress,” “O_3_,” and “salinity” and the name of a particular vegetable were also used as keywords. The references cited in the obtained references were also collected. Strawberry, which is categorized as a vegetable in some countries, and potato, which is considered a vegetable as it is rich in ascorbic acid but regarded as staple food due to the large amount of accumulated starch, were included in the database search. The vegetables were classified as root vegetables, stem vegetables, leafy vegetables, and fruit vegetables. Root vegetables included carrot, radish, sugar beet, and turnip; stem vegetables included broccoli, celery, celtuce, Chinese kale, ginger, onion, potato, and scallion; leafy vegetables included cabbage, Chinese cabbage, chives, fenugreek, Hongfengcai, lettuce, oily sowthistle, palak, and spinach; fruit vegetables included cucumber, hot pepper, strawberry, sweet pepper, and tomato. As the common names of several vegetables are not commonly known worldwide, their Latin names are shown here: Chinese kale (*Brassica oleracea* L. var. alboglabra), fenugreek (*Trigonella foenum-graecum* L.), hongfengcai or guanyinxian (*Gynura bicolor* L.), oily sowthistle (*Sonchus oleraceus* L.), palak (*Beta vulgaris* L. var. allgreen), and celtuce (*Lactuca sativa* L. var. augustana).

Fifty-six journal articles and one conference article published in English and meeting the following criteria were included in this analysis: (1) the ambient CO_2_ for plant growth (rather than storage) was ≥200 and ≤450 μmol^-1^, while that of eCO_2_ was between 540 and 1200 μmol^-1^; (2) measurements of nutritional quality of vegetables were collected, including soluble sugar, organic acid, protein, nitrate, antioxidants, and minerals (see Tables in **Supplementary Information**). If a study involved several species, cultivars, growth stages, or was conducted for several years or under various CO_2_ levels, all the observations were regarded to be independent and included in the database. The data extracted from each study were the means and the replicate number of the measurements under both ambient CO_2_ and eCO_2_. For the values that cannot be directly extracted from tables and text, i.e., data in figures, the height of the columns in figures was measured to estimate the observation using ImageJ (version 1.51a, National Institutes of Health, United States).

### Statistical Analysis

The significant level for comparing the effect of CO_2_ on nutritional quality, shown in Tables in **Supplementary Information**, was at *p* < 0.05. If no multiple comparisons were performed in the references, a two-tailed *t*-test was used to indicate the significance of the effect of eCO_2_ on vegetable quality based on the means, standard error/deviation, and the number of replicates using Microsoft Excel 2016. If only the minimal and maximal values (i.e., the range) of the treated CO_2_ concentration were given in a study, the treated CO_2_ concentration was estimated as their average, making it possible to calculate the ratio of concentration of eCO_2_ to ambient CO_2_ (Tables in **Supplementary Information**).

A meta-analysis was conducted to assess the effect of CO_2_ on vegetable quality on the well-reported variables, i.e., concentrations of fructose, glucose, sucrose, total soluble sugar, titratable acidity, total protein, nitrate, total antioxidant capacity, total phenols, total flavonoids, ascorbic acid, total chlorophyll, chlorophyll a, chlorophyll b, carotenoids, lycopene, anthocyanins, phosphorus (P), potassium (K), calcium (Ca), magnesium (Mg), sulfur (S), iron (Fe), manganese (Mn), copper (Cu), and zinc (Zn), following the methods described by Wang et al. (2012). The effect size metric was the response ratio:

R=E/A

where R, E, and A are the response ratio, and the means of quality response under eCO_2_ and ambient CO_2_, respectively. The technique of the natural logarithm-transformed ratio (ln R) was used for analysis to reduce biases toward increases (Hedges et al., 1999; Jablonski et al., 2002). Meta-analytic studies normally weigh the effect size by the reciprocal of their variance, which gives greater weight to experiments with greater precision. The effect size can also be weighted by the number of replicates (van Groenigen et al., 2011; Lam et al., 2012; Loladze, 2014). However, many eCO_2_ studies did not report the variation and sample size of the measurements. Therefore, the unweighted method (Loladze, 2014) was used in this work. The means and 95% confidence intervals of effect size were determined using the nonparametric bootstrap method (5,000 iterations) using the package bootES in R software (version 3.3.2) (Kirby and Gerlanc, 2013). The CO_2_ effect was considered as significant when the 95% confidence intervals did not overlap with zero. The effect size was back-transformed to ordinary percentage change to ease interpretation.

The potential publication bias in the meta-analysis was assessed based on the correlation of effect size (ln R) and the sample sizes/replicates for each study and each measurement regardless of the observations without reporting the number of replicates (Wang et al., 2012; Loladze, 2014). The funnel-shaped and symmetrical cloud of points (**Supplementary Figure S2**) indicates the absence of any significant publication bias (Egger et al., 1997).

## Results and Discussion

### Effect of eCO_2_ on Soluble Sugar and Acidity

**FIGURE 1:**
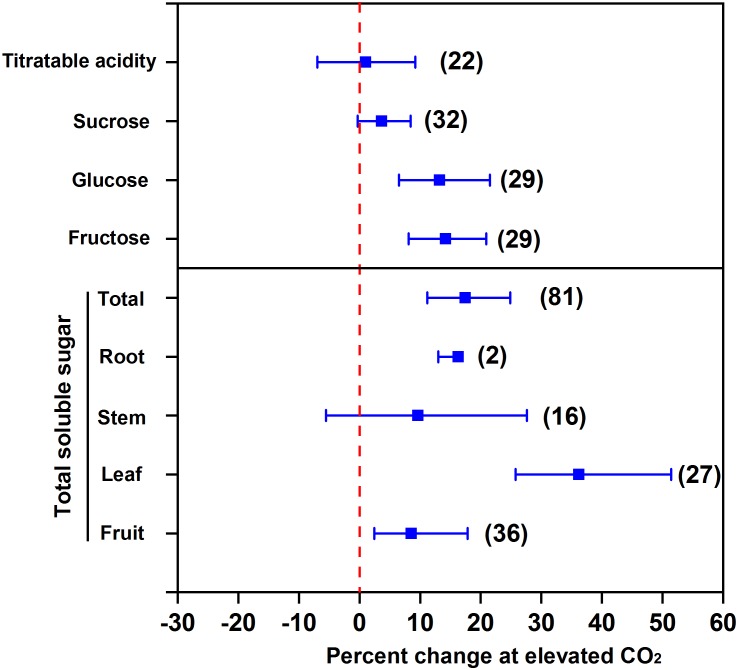
Effect of elevated CO_2_ on the concentrations of soluble sugar and acidity in vegetables. Data are means of percent change with 95% confidence intervals (indicated with error bars) under elevated CO_2_ compared to ambient CO_2_. The number of observations is in parentheses.

Elevated CO_2_ promotes soluble sugar accumulation in the edible parts of vegetables. The increased CO_2_ fixation under eCO_2_ promotes the synthesis of triose phosphate in leaves (Long et al., 2004), which can be further transformed into other carbohydrates, e.g., glucose, fructose, and sucrose. Our meta-analysis showed that eCO_2_ increased the concentrations of glucose by 13.2%, fructose by 14.2%, sucrose by 3.7% (at *p* = 0.07), and total soluble sugar by 17.5% in terms of all vegetables (**Figure 1**). The increment of total soluble sugar in leaf (an organ for carbohydrate synthesis) under eCO_2_ was the greatest (36.2%) among all the classes of vegetables. The increment can reach 38–188% in the leaves of Chinese cabbage and 16–53% in the leaves of oily sowthistle (Jin et al., 2009). Compared to leafy vegetables, the increments of total soluble sugar were less in fruit and root vegetables, and were 8.5% and 16.3%, respectively. This indicates that the synthesized carbohydrates in leaves cannot be fully translocated to fruits as well as to roots, although one needs to be cautious regarding the species variation. For example, eCO_2_ (950 μmol mol^-1^) increased total soluble sugar in strawberry fruits by 20% relative to 350 μmol mol^-1^ (Wang and Bunce, 2004). Similarly, the total soluble sugar was increased by 13% in radish and 20% in turnip under 1,000 μmol mol^-1^ CO_2_ compared to 400 μmol mol^-1^ control (Azam et al., 2013).

However, eCO_2_ does not affect the total soluble sugar in stem vegetables (**Figure 1**). It is possible that eCO_2_ promotes the transformation of soluble sugar to lignin, which counteracts the carbon transformation toward soluble sugar accumulation (Liu et al., 2018). Elevated CO_2_ was found to even decrease soluble sugar concentration in stem vegetables, e.g., celery (Jin et al., 2009). Likewise, we found that eCO_2_ had no effect on titratable acidity (**Figure 1**), which indicates that eCO_2_ promotes the transformation of fixed CO_2_ to soluble sugar to a greater extent than that to organic acids (Wang and Bunce, 2004) and thus allows a greater sugar-to-acid ratio and a stronger taste of vegetables.

### Effect of eCO_2_ on Nitrogenous Compounds

**FIGURE 2:**
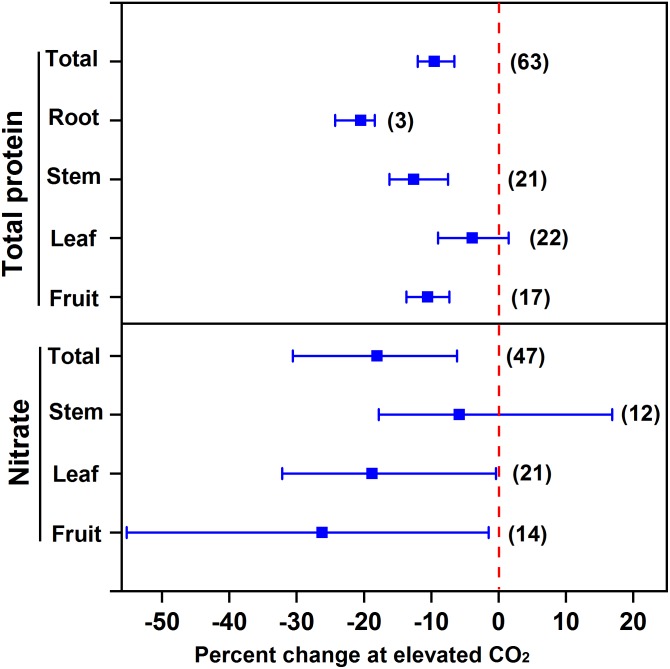
Effect of elevated CO_2_ on the concentrations of total protein and nitrate in vegetables. Data are means of percent change with 95% confidence intervals (indicated with error bars) under elevated CO_2_ compared to ambient CO_2_. The number of observations is in parentheses.

Our meta-analysis showed that eCO_2_ decreased the protein concentration in vegetables (9.5%), specifically 10.5% for fruit vegetables, 12.6% for stem vegetables, and 20.5% for root vegetables (**Figure 2**). However, no significant effect was observed for leafy vegetables. Since leafy vegetables generally contain a greater concentration of nitrate, eCO_2_ may promote N assimilation in leaves (Stitt and Krapp, 1999). For example, eCO_2_ increased the N concentration in the inner leaves of lettuce cv. “Batavia Rubia Munguía” noninoculated with arbuscular mycorrhizal fungi to a greater extent than the outer leaves (Baslam et al., 2012). Moreover, eCO_2_ limits the uptake of nitrogen and the synthesis of nitrogenous compounds of vegetables to a lesser extent than that of other crops (mainly grain crops) (9.5% vs. 10–15%) (Taub et al., 2008; Loladze, 2014), probably because N deficiency is more common for grain crop cultivation in soils compared to vegetable cultivation.

On the other hand, eCO_2_ may alleviate the potential toxicity from nitrate intake for human beings. Overall, eCO_2_ decreased the nitrate concentration of all vegetables by 18.0% (**Figure 2**). Specifically, eCO_2_ decreased the nitrate concentration in fruit and leafy vegetables by 26.2% and 18.8%, respectively. This indicates that eCO_2_ promotes the nitrate assimilation to a greater extent than nitrate uptake. Bloom et al. (2010) found that eCO_2_ inhibited nitrate assimilation of wheat and Arabidopsis initially and then reduced nitrate uptake, which further limited N assimilation progressively. The big variation in the effect of eCO_2_ on nitrate concentration might be due to its distinct impact among species. For example, eCO_2_ could have greatly decreased the nitrate concentration in cucumber (Tang et al., 2018), but greatly increased that in tomato (Wei et al., 2018). This is probably why the overall eCO_2_ effect on stem vegetables was not significant. Elevated CO_2_ was found to sharply increase the nitrate accumulation in celtuce but decrease that in celery (Jin et al., 2009).

Furthermore, it appears that eCO_2_ affected the components of free amino acid in lettuce (Miyagi et al., 2017), potato (Högy and Fangmeier, 2009), and sweet pepper (Piñero et al., 2017a,b). This suggests that eCO_2_ has different effects on the metabolic process of amino acids, whose mechanisms are unclear till now.

### Effect of eCO_2_ on Antioxidants

**FIGURE 3:**
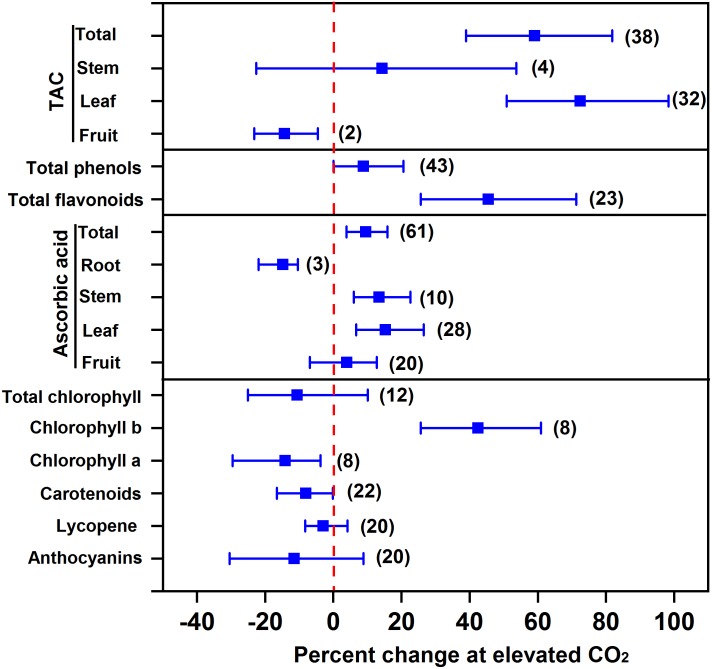
Effect of elevated CO_2_ on the concentrations of antioxidants in vegetables. Data are means of percent change with 95% confidence intervals (indicated with error bars) under elevated CO_2_ compared to ambient CO_2_. The number of observations is in parentheses. TAC, total antioxidant capacity.

Overall, eCO_2_ promotes the accumulation of antioxidants in vegetables, thus improving vegetable quality. Our results showed that eCO_2_ increased total antioxidant capacity, total phenols, total flavonoids, ascorbic acid, and chlorophyll b by 59.0%, 8.9%, 45.5%, 9.5%, and 42.5%, respectively, indicating an improvement of beneficial compounds in vegetables (**Figure 3**). The greatest increase in total antioxidant capacity (72.5%) as well as the greatest increase in ascorbic acid (15.3%) were both observed in leafy vegetables among different types of vegetables (**Figure 3**). It is reasonable to predict that the increased soluble sugar as precursors can increase the synthesis and accumulation of antioxidants (Wang et al., 2003; Jaafar et al., 2012; Becker and Kläring, 2016). For example, compared to 200 μmol mol^-1^ CO_2_ control, 1,000 μmol mol^-1^ CO_2_ is supposed to promote sugar accumulation and subsequently phenol synthesis in lettuce leaf (Becker and Kläring, 2016). On the other hand, eCO_2_ is thought to promote NADPH synthesis, such that eCO_2_ can enhance the plants’ capability of diverting NADPH to maintain a higher concentration of antioxidants, e.g., glutathione and ascorbate, to counteract damage from ozone (O_3_) (Rao et al., 1995) or other stresses (Xu et al., 2015).

In contrast, eCO_2_ had no significant effect on the accumulation of pigments like total chlorophyll, carotenoids, lycopene, and anthocyanins, and even decreased the concentration of total antioxidant capacity in fruit vegetables, ascorbic acid in root vegetables, chlorophyll a, and carotenoids by 14.4%, 14.8%, 14.1%, and 8.1%, respectively. Elevated CO_2_ can decrease the photorespiration of plants when grown in high light intensity. This may consequently decrease the formation of oxygen radicals, thus reducing antioxidant metabolism (Pérez-López et al., 2018). A recent study on the transcript profile of genes in carrots found that eCO_2_ could affect antioxidant accumulation (i.e., ascorbic acid) through a complex process, involving the synthesis, recycling, and degradation of ascorbic acid (Wu et al., 2017).

### Effect of eCO_2_ on Minerals

**FIGURE 4:**
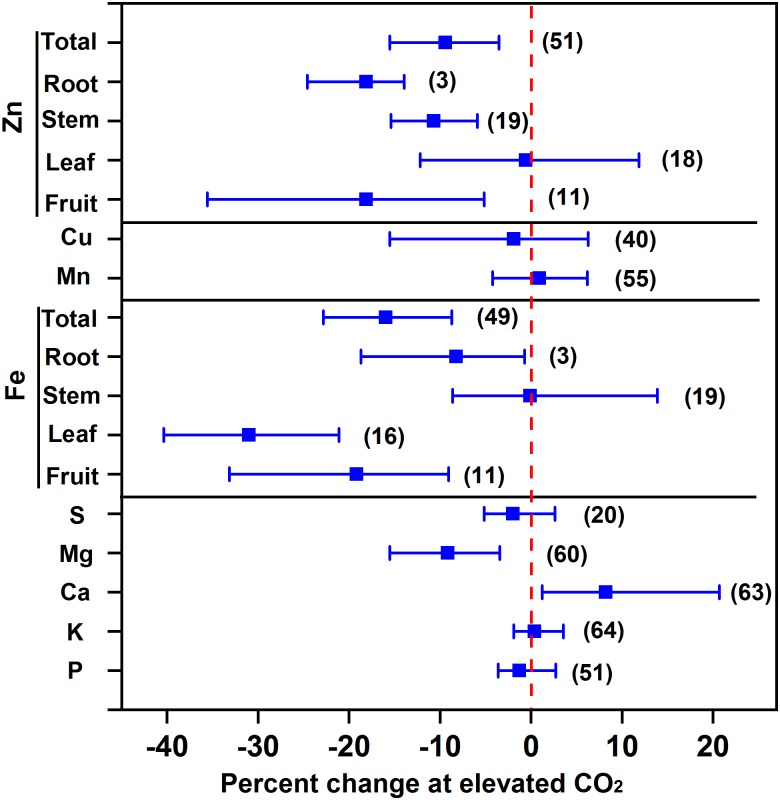
Effect of elevated CO_2_ on the concentrations of minerals in vegetables. Data are means of percent change with 95% confidence intervals (indicated with error bars) under elevated CO_2_ compared to ambient CO_2_. The number of observations is in parentheses.

Our results showed that eCO_2_ decreased the concentrations of Mg, Fe, and Zn by 9.2%, 16.0%, and 9.4%, respectively, whilst it maintained the concentrations of P, K, S, Cu, and Mn of vegetables as a whole (**Figure 4**). The decrease in Fe concentration was the greatest in leafy vegetables (31.0%), followed by fruit vegetables (19.2%) and root vegetables (8.2%), whereas the decrease in Zn concentration was 18.1% in both fruit and root vegetables and 10.7% in stem vegetables. The decrease in Fe was greater than that in wheat (5.1%) and rice (5.2%) (Myers et al., 2014) or in C_3_ plants (10%) (Loladze, 2014). As Fe and Zn are important for human nutrition, particularly for children (Myers et al., 2014), their deficiency in vegetables under future CO_2_ climates should not be neglected.

Several studies reported that eCO_2_ decreased the mineral concentration by a dilution effect (Fangmeier et al., 2002; Högy and Fangmeier, 2009; Loladze, 2014) or restricted transpiration (McDonald et al., 2002). For example, recent reviews demonstrated that eCO_2_ decreased all the mineral concentrations in grain crops (Loladze, 2014; Myers et al., 2014), suggesting that the decrease in mineral concentration is not specifically regulated by certain metabolic processes but by a dilution effect due to the increased biomass. However, **Figure 4** shows that the Ca concentration of vegetables was increased by 8.2% under eCO_2_, indicating that neither the dilution effect nor the limitation of transpiration can explain the greater accumulation of Ca in vegetables. The inhibition of fruit enlargement resulting from other environmental factors (e.g., N deficiency) may increase Ca storage in fruits (Dong et al., 2018; Tang et al., 2018).

### Interactions of eCO_2_ With Other Factors

As mentioned above, the effects of eCO_2_ on vegetable quality vary among various types of vegetables and environments. However, limited data are available for a robust meta-analysis on the interaction of eCO_2_ with several factors. Therefore, we qualitatively summarize the limited studies here involving the following factors: plant cultivar, CO_2_ level, growth stage, light, O_3_ stress, nutrient, and salinity.

#### Plant Cultivar

Elevated CO_2_ generally increased the total flavonoids in some onion cultivars with a greater ability of flavonoid accumulation like cv. “Choesty” but had no effect on other cultivars with less constitutive flavonoid accumulation like cv. “Fineleaf” (Thompson et al., 2004). Elevated CO_2_ potentially promoted the accumulation of antioxidative compounds to a greater extent in cultivars richer in the constitutive accumulation of antioxidants (Pérez-López et al., 2015a). By contrast, eCO_2_ enhanced the glycoalkaloid concentration in potato cv. “Russet Burbank” to a greater extent than cv. “Norland,” but their abilities of producing glycoalkaloid were similar (Nitithamyong et al., 1999). Elevated CO_2_ led to a greater nitrate accumulation in the leaves of pigmented lettuce cv. “Blonde of Paris Batavia” in nonsaline conditions and decreased that in the leaves of cv. “Oak Leaf,” which possesses a similar ability of nitrate accumulation (Pérez-López et al., 2015a). CO_2_ effects on mineral concentrations can differ between lettuce cultivars (cv. “Batavia Rubia Munguía” vs. cv. “Maravilla de Verano”) (Baslam et al., 2012). In general, the constitutive differences of compound accumulation in tissues might contribute to the intraspecific variation in response to eCO_2_.

#### CO_2_ Level

Higher CO_2_ results in greater photosynthetic rates initially but often photosynthetic acclimation, i.e., down regulation of net photosynthetic rates in the long term (Long et al., 2004; Kirschbaum, 2011), which indicates that increases in CO_2_ concentration can substantially affect vegetable quality. Elevated CO_2_ (by 600 μmol mol^-1^ above ambient CO_2_) enhanced the concentrations of fructose, glucose, and sucrose, but reduced the concentrations of malic acid, citric acid, and quinic acid in strawberry fruits to a greater extent than an elevation of CO_2_ of 300 μmol mol^-1^ (Wang and Bunce, 2004), indicating greater carbohydrate accumulation and less organic acid transformation from carbohydrates in strawberry under higher CO_2_ concentration. On the other hand, compared to ambient CO_2_, a lower CO_2_ elevation (550 μmol mol^-1^) increased the concentrations of fructose and glucose in potato at maturity to a greater extent than a higher CO_2_ elevation (680 μmol mol^-1^) (Högy and Fangmeier, 2009), indicating an inhibition of carbohydrate synthesis under long-term higher CO_2_ exposure. Interestingly, the reverse was true for total glycoalkaloid concentration in potato fruits (Högy and Fangmeier, 2009), implying that the effect of CO_2_ on carbon metabolism varies with the direction of shifts. Similarly, 550 μmol mol^-1^ CO_2_ decreased the concentrations of phenols and flavonoids, and the total antioxidant capacity of tomato fruits to a greater extent than 700 μmol mol^-1^, but it increased the concentration of ascorbic acid to a greater degree than that under 700 μmol mol^-1^ (Mamatha et al., 2014). Differences in product quality among different CO_2_ levels were also reported in other studies (Levine and Paré, 2009; Ren et al., 2014; Fu et al., 2015).

#### Growth Stage

Elevated CO_2_ had increased the sucrose concentration of tomato fruits to a greater extent at the early fruiting stage than that at the later fruiting stage (Islam et al., 1996). The same pattern was noticed in terms of the concentrations of soluble sugars and organic acids in grapes (Bindi et al., 2001). The minimal impact on sugar accumulation at the later growth stage may be attributed to the long-term CO_2_ exposure when less carbon can be fixed and translocated to fruits. Tomato fruits are usually harvested when their color meets certain standards (Zhang et al., 2014). However, eCO_2_ has been found to increase the synthesis of color-related pigments in tomatoes to a lesser extent than the synthesis of soluble sugar and total solids (Islam et al., 1996; Zhang et al., 2014), resulting in a mismatch of the fruit color with its maturity. This means that producers may have delayed harvesting the fruits under eCO_2_ based on the standards set for ambient CO_2_. Elevated CO_2_ thus increases soluble sugar accumulation as the sugar concentration is continuously increasing from the green to the red stage of tomato (Winsor et al., 1962). This might also explain the decrease in the concentrations of organic acids (Islam et al., 1996) and ascorbic acid (Khan et al., 2013) in tomato fruits at the red stage under eCO_2_, because the concentrations of organic acids and ascorbic acid were generally increased when tomato reaches its maturity but decreased after ripening (Islam et al., 1996). Elevated CO_2_ might promote soluble sugar and fiber accumulation in tomato cv. “Eureka” by accelerating its maturity as well (Khan et al., 2013).

Elevated CO_2_ generally increased N concentration in the leaves of spinach and fenugreek at 40 days after CO_2_ exposure, but N concentration declined at 80 days after CO_2_ exposure (Jain et al., 2007), indicating that eCO_2_ probably promotes N assimilation of these leafy vegetables at the early stage and this is consistent with a study on barley seedlings (Robredo et al., 2011), but N assimilation is limited due to photosynthetic acclimation at the later stage (Taub et al., 2008).

#### Light

Plants grown in moderate vs. low light can generate more ATP and NADPH for carbon fixation, whereas high vs. moderate light may cause photoinhibition resulting from excessive light intensity that produces greater amounts of reactive oxygen species (Pérez-López et al., 2018). Elevated CO_2_ can improve vegetable quality under certain light intensities. For example, both eCO_2_ (800 μmol mol^-1^) and high light intensity (800 μmol m^-2^ s^-1^) decreased nitrate accumulation in spinach leaves independently and interactively, resulting in the lowest nitrate concentration in the combination of eCO_2_ with high light intensity (Proietti et al., 2013). A combination of eCO_2_ (700 vs. 400 μmol mol^-1^) and high light intensity (700 vs. 400 μmol m^-2^ s^-1^) increased the total antioxidant capacity of both cultivars of lettuce relative to ambient CO_2_ and high light intensity (Pérez-López et al., 2015b). However, this combination could decrease the total antioxidant capacity in both cultivars observed in another study with the same growth conditions (Pérez-López et al., 2018), indicating that the total antioxidant capacity can be very sensitive to environmental factors. Similarly, eCO_2_ decreased the concentrations of ascorbic acid and capsaicin to a greater extent under high light intensity (463 vs. 233 μmol m^-2^ s^-1^) (Li et al., 2017). In terms of light quality, increases in CO_2_ concentration (from 450 to 1200 μmol mol^-1^) generally improved the concentration of phenols, anthocyanin, and flavonoids in RB20 (ratio of red LED light to blue light, 80%/20%) to a greater extent than that in RB40 (red/blue, 60%/40%), with the same light intensity at 250 μmol m^-2^ s^-1^ (Ren et al., 2014). Compared to ambient CO_2_ without ultraviolet-B radiation, the combination of eCO_2_ (700 vs. 350 μmol mol^-1^) with ultraviolet-B radiation enhanced the concentrations of soluble sugar, ascorbic acid, and lycopene in tomato fruits (to be the best for ultraviolet-B radiation at 1.2 kJ m^-2^) (Li et al., 2007).

#### O_3_ Stress

Increases in ground-level O_3_ also contribute to global warming and other aspects of environmental change (IPCC, 2014). O_3_ is easily absorbed through plant stomata, and it induces the formation of reactive oxygen species and free radicals damaging the components of plant cells and inhibits plant growth (Kumari et al., 2013). Elevated CO_2_ may alleviate the adverse effect of O_3_ through decreasing stomatal conductance, and thus O_3_ uptake (Kumari et al., 2013). Therefore, under O_3_-stressed conditions, eCO_2_ promotes carbon fixation and improves the downstream metabolic process related to vegetable quality, e.g., promoting carbohydrate synthesis, including soluble sugar in the leaves of palak (Kumari et al., 2013) and tubers of potato (Kumari and Agrawal, 2014). In addition, eCO_2_ can reduce the chlorophyll destruction in leaves under O_3_ stress and enhance the activity of antioxidant systems in the leaves of palak, including activities of ascorbate peroxidase (Kumari et al., 2013). By contrast, the concentrations of K, Ca, Na, Fe, and Zn in tubers of potato decreased under eCO_2_ and high O_3_ relative to ambient CO_2_ and ambient O_3_ (Kumari and Agrawal, 2014), thereby decreasing tuber quality. The interaction between eCO_2_ and O_3_ has not been detected in quality-related parameters, e.g., soluble sugar, organic acid, and ascorbic acid, in another study on potato (Donnelly et al., 2001).

#### Nutrient

Nutrient availability also influences the effect of eCO_2_ on product quality. Low nutrient availability limits the eCO_2_ effect on plant photosynthetic rates (Arp, 1991; Gruda and Tanny, 2015), probably resulting in less carbon available for synthesizing secondary compounds. For example, eCO_2_ reduced the concentrations of soluble solids, soluble sugar, and lycopene in tomato fruits in normal N availability where less carbon was fixed, while it promoted their concentration in higher N availability (Helyes et al., 2012). Similarly, eCO_2_ decreased the total antioxidant capacity and antioxidant compounds of strawberry in low N availability to a greater extent than that in high N (Sun et al., 2012). However, eCO_2_ increased the concentrations of aliphatic glucosinolates of Chinese kale under N supply at 5 and 10 mmol L^-1^ N, but decreased their concentrations at 20 mmol L^-1^ N with unknown mechanisms (La et al., 2009). On the other hand, low nutrient availability exacerbates the uptake and assimilation of the nutrient itself under eCO_2_, as evidenced by a greater decrease in grain protein concentration under low N availability (Taub et al., 2008). The trend is also true for vegetables. For example, eCO_2_ decreased the total N concentration in the bolting stem of Chinese kale in high N supply to a lesser extent than that in low N (La et al., 2009).

Interestingly, N form also interacts with eCO_2_ and hence affects the product quality of Chinese cabbage (Reich et al., 2016) and sweet pepper (Piñero et al., 2017b). Specifically, eCO_2_ (800 vs. 420 μmol mol^-1^) increased P concentration and maintained S concentration in shoots of 20-day-old Chinese cabbage in 1.88 mM NH_4_NO_3_ at a temperature of 15/12°C (day/night), while it maintained P concentration and decreased S concentration in plants supplied with 3.75 mM NO_3_^-^ (Reich et al., 2016). Elevated CO_2_ generally increased the concentrations of chlorophyll a, chlorophyll b, protein, P, Ca, Mg, Mn, Cu, and cadaverine in fruits of sweet pepper when supplied with 10 mM NO_3_^-^ and 2 mM NH_4_^+^, but decreased these parameters under 12 mM NO_3_^-^ (Piñero et al., 2017b). Although NH_4_^+^ can be toxic when supplied at high levels for vegetable growth (Schjoerring et al., 2002; Roosta and Schjoerring, 2008), eCO_2_ appears to improve the nutritional quality of vegetables when supplied N source partly using NH_4_^+^.

#### Salinity Stress

Saline water (such as 5 dS m^-1^) is widely used in greenhouse vegetable cultivation in some countries like Israel and there are interactive effects between eCO_2_ and salinity on vegetable quality (Mizrahi and Pasternak, 1985; Li et al., 1999). Elevated CO_2_ increased the productivity and yield of tomato under saline conditions (7 dS m^-1^); however, the quality parameters—total soluble sugar, total soluble solids, and acidity—remained stable (Li et al., 1999). In contrast, eCO_2_ increased nitrate accumulation in the leaves of pigmented lettuce cv. “Blonde of Paris Batavia” in saline conditions (200 mmol L^-1^ NaCl) to a lesser extent than that in nonsaline conditions (Pérez-López et al., 2015a), thus benefiting lettuce quality.

Elevated CO_2_ reduced anthocyanin synthesis in the leaves of pigmented lettuce cv. “Oat leaf” to a greater extent in saline conditions than nonsalinity (Pérez-López et al., 2015a). However, eCO_2_ tended to increase the concentration of reduced ascorbate in cv. “Oat leaf” under saline conditions (Pérez-López et al., 2015a). Therefore, eCO_2_ might enhance the synthesis of some components of antioxidants rather than decreasing the concentrations of all antioxidants (**Figure 3**). The increase in total antioxidant activity under eCO_2_ was also observed under saline conditions for both lettuce cultivars in another study (Pérez-López et al., 2013).

Apart from the above factors, the interactive effect of eCO_2_ with temperature (Sun et al., 2012) and water availability (Wei et al., 2018) has also been investigated.

### Recommendations for Optimizing eCO_2_ Benefits

Several approaches can be considered to combine eCO_2_ with other factors to enhance the nutritional quality of vegetables: (1) selecting vegetable species or cultivars that possess greater ability in carbon fixation and synthesis of required quality-related compounds; (2) optimizing other environmental factors (e.g., moderate CO_2_ concentrations, moderate light intensity, increased N availability, or increased fertilization of Fe or Zn) to promote carbon fixation and nutrient uptake interactively when growing plants under eCO_2_; (3) harvesting vegetable products earlier in cases of over maturity and reduced benefit of eCO_2_ to vegetative growth; and (4) combining eCO_2_ with mild environmental stress (e.g., ultraviolet-B radiation or salinity) in instances when this enhances vegetable quality and might counteract the dilution effect or direct metabolic pathways toward the synthesis of health-beneficial compounds. However, one needs to be cautious that it is less likely to improve all the parameters of nutritional quality simultaneously. An improvement of quality might result in yield penalty.

### Effects of Elevated CO_2_ on Quality of Some Specific Crops

#### Lettuce

Lettuce is one of the most preferred vegetables worldwide for its taste, flavor, and richness in healthy compounds. Therefore, the effects of eCO_2_ and its interactions with other factors on vegetable quality have received more attention in lettuce than other vegetables (Pérez-López et al., 2013, 2015a,b, 2018; Becker and Kläring, 2016; Sgherri et al., 2017). Elevated CO_2_ potentially enhances the taste of lettuce indicated by increasing soluble sugar accumulation by 27.1% (*n* = 18, meta-analysis), as demonstrated in several studies (Jin et al., 2009; Baslam et al., 2012; Pérez-López et al., 2015a; Becker and Kläring, 2016). More specifically, eCO_2_ increased the soluble sugar concentration in the outer layer of the leaves of cv. “Maravilla de Verano” to a greater extent than that of the inner layer (Baslam et al., 2012), whereas there was no similar promotion by eCO_2_ if plants were grown in a high light intensity of 700 μmol m^-2^ s^-1^ (Pérez-López et al., 2015b). The protein concentration was decreased by 5.6% under eCO_2_ (*n* = 12, meta-analysis at *p* = 0.07), indicating a decreased nutritional value. By contrast, the meta-analytic results showed that eCO_2_ increased ascorbic acid and total antioxidant capacity by 7.1% (*n* = 18) and 82.0% (*n* = 23), respectively, indicating an improvement of lettuce quality. However, the responses of healthy compounds in lettuce to eCO_2_, including phenolic acid, flavonoid, ascorbic acid, and pigments, are not consistent among growth conditions or studies (**Supplementary Table S4**). For example, eCO_2_ greatly promoted ascorbic acid concentration (Jin et al., 2009), but not always (Baslam et al., 2012; Pérez-López et al., 2015a,b). In general, (1) lettuce plants received more benefits from eCO_2_ on its antioxidant metabolism under salinity stress or high light intensity (Pérez-López et al., 2013, 2015a) and (2) eCO_2_ promoted the accumulation of antioxidative compounds in cv. “Oak Leaf” to a greater extent than that of cv. “Blonde of Paris Batavia” (Pérez-López et al., 2015a,b).

#### Tomato

The effect of eCO_2_ on tomato fruit quality has also received much attention in research. Our analysis indicates that eCO_2_ enhances tomato quality, and likely taste, by increasing the concentrations of fructose, glucose, and total soluble sugars (14.7% using meta-analysis, *n* = 24) in tomato fruits (Islam et al., 1994; Behboudian and Tod, 1995; Li et al., 2007; Zhang et al., 2014). This effect was reduced for fruits of the first several harvests (Islam et al., 1994; Zhang et al., 2014) and under moderate N supply (Helyes et al., 2012) when fewer carbohydrates were accumulated. Compared with soluble sugar, total soluble solids and organic acids were increased to a lesser extent (Behboudian and Tod, 1995; Zhang et al., 2014), probably due to the less transformation from increased sugar. Generally, eCO_2_ increased the concentration of ascorbic acid by 18.5% (*n* = 12, meta-analysis). In contrast, the effect of eCO_2_ on lycopene concentration is variable (**Supplementary Table S4**), perhaps due to the sensitivity of lycopene synthesis to temperature (Krumbein et al., 2006), and thus our meta-analysis (*n* = 18) found no significant effect of eCO_2_ on lycopene concentration. Together, these results indicate that more research on the interactive effects of eCO_2_ and other growth conditions on tomato fruit quality is needed.

#### Potato

The program **Ch**anging Climate and Potential **I**mpacts on **P**otato Yield and Quality (CHIP) funded by the European Commission pursues a comprehensive exploration of the effects of eCO_2_ on potato quality (De Temmerman et al., 2002). Their results showed that the eCO_2_ effect on the nutritional and processing quality of potato can be variable (Högy and Fangmeier, 2009). Elevated CO_2_ increased the concentrations of soluble sugars and starch, and maintained the concentrations of organic acids when the growth condition is suitable for a greater yield in general, resulting in a higher risk of browning and increased acrylamide production when fried (Donnelly et al., 2001; Kumari and Agrawal, 2014). Otherwise, the reducing sugar concentration can be decreased (Kumari and Agrawal, 2014). The decreased citrate concentration led to a higher risk of discoloration but resulted in better taste (Högy and Fangmeier, 2009). The protein and Zn concentration in tubers was lower (13.1%, *n* = 18 and 10.7%, *n* = 19, respectively, from meta-analysis) under eCO_2_ and thus reduced the nutritive value of tubers as shown in several studies (Donnelly et al., 2001; Fangmeier et al., 2002; Heagle et al., 2003; Högy and Fangmeier, 2009) except when eCO_2_ recovered plant growth from intense stresses (Kumari and Agrawal, 2014). The total glycoalkaloid and α-chaconine concentration under eCO_2_ was decreased (Vorne et al., 2002; Högy and Fangmeier, 2009), remained stable (Donnelly et al., 2001), or increased (Nitithamyong et al., 1999). The judgment of the glycoalkaloids also differs as their decreases can be regarded as positive in terms of its toxicity but as negative in terms of a worse taste (Högy and Fangmeier, 2009). In conclusion, potato quality under eCO_2_ generally should be assessed in terms of the corresponding parameters and the needs of customers.

## Summary

Several studies have been conducted in recent decades on the effects of eCO_2_ on vegetable quality, including parameters related to taste, flavor, nutritive value, and industrial processing. These studies show that eCO_2_ can promote the accumulation of soluble sugar including glucose and fructose, and the accumulation of antioxidants including ascorbic acid, total phenols, and total flavonoids, but reduce the levels of protein, nitrate, Mg, Fe, and Zn in products. In practice, it is advisable to enhance vegetable quality by (1) selecting species or cultivars that respond well to eCO_2_; (2) providing optimal environments together with eCO_2_; (3) harvesting vegetables earlier than standards set at ambient CO_2_; and (4) combining with moderate environmental stresses. The promotion by the increased carbon fixation and thus the precursor, dilution effect, stress induction, and limitation by transpiration or N assimilation can generally explain the shifts of vegetable quality under eCO_2_. However, research is still required to reveal the underlying physiological and molecular mechanisms more specifically.

## Author Contributions

JD, NG, and XL conceived and designed the review structure. JD collected references, analyzed the data, and wrote the paper. NG, XL, SL, and ZD revised the paper.

## Conflict of Interest Statement

The authors declare that the research was conducted in the absence of any commercial or financial relationships that could be construed as a potential conflict of interest.

## Footnotes

1 https://www.co2.earth

## References

[B1] Arp W. (1991). Effects of source-sink relations on photosynthetic acclimation to elevated CO_2_. *Plant Cell Environ.* 14 869–875. 10.1111/j.1365-3040.1991.tb01450.x

[B2] Azam A. Khan I. Mahmood A. Hameed A. (2013). Yield, chemical composition and nutritional quality responses of carrot, radish and turnip to elevated atmospheric carbon dioxide. *J. Sci. Food Agric.* 93 3237–3244. 10.1002/jsfa.6165 23576218

[B3] Baslam M. Garmendia I. Goicoechea N. (2012). Elevated CO_2_ may impair the beneficial effect of arbuscular mycorrhizal fungi on the mineral and phytochemical quality of lettuce. *Ann. Appl. Biol.* 161 180–191. 10.1111/j.1744-7348.2012.00563.x

[B4] Becker C. Kläring H.-P. (2016). CO_2_ enrichment can produce high red leaf lettuce yield while increasing most flavonoid glycoside and some caffeic acid derivative concentrations. *Food Chem.* 199 736–745. 10.1016/j.foodchem.2015.12.059 26776031

[B5] Behboudian M. H. Tod C. (1995). Postharvest attributes of ‘Virosa’ tomato fruit produced in an enriched carbon dioxide environment. *Hortic. Sci.* 30 490–491.

[B6] Bindi M. Fibbi L. Miglietta F. (2001). Free air CO_2_ enrichment (FACE) of grapevine (*Vitis vinifera* L.): II. Growth and quality of grape and wine in response to elevated CO_2_ concentrations. *Eur. J. Agron.* 14 145–155. 10.1016/S1161-0301(00)00093-9

[B7] Bisbis M. B. Gruda N. Blanke M. (2018). Potential impacts of climate change on vegetable production and product quality–a review. *J. Clean. Prod.* 170 1602–1620. 10.1016/j.jclepro.2017.09.224

[B8] Bloom A. J. Burger M. Asensio J. S. R. Cousins A. B. (2010). Carbon dioxide enrichment inhibits nitrate assimilation in wheat and *Arabidopsis*. *Science* 328 899–903. 10.1126/science.1186440 20466933

[B9] De Temmerman L. Hacour A. Guns M. (2002). Changing climate and potential impacts on potato yield and quality ‘CHIP’: introduction, aims and methodology. *Eur. J. Agron.* 17 233–242. 10.1016/S1161-0301(02)00063-1

[B10] Demmers-Derks H. Mitchell R. A. C. Mitchell V. J. Lawlor D. W. (1998). Response of sugar beet (*Beta vulgaris* L.) yield and biochemical composition to elevated CO_2_ and temperature at two nitrogen applications. *Plant Cell Environ.* 21 829–836. 10.1046/j.1365-3040.1998.00327.x

[B11] Dong J. Xu Q. Gruda N. Chu W. Li X. Duan Z. (2018). Elevated and super-elevated CO_2_ differ in their interactive effects with nitrogen availability on fruit yield and quality of cucumber. *J. Sci. Food Agric.* 10.1002/jsfa.8976 [Epub ahead of print]. 29479715

[B12] Donnelly A. Lawson T. Craigon J. Black C. R. Colls J. J. Landon G. (2001). Effects of elevated CO_2_ and O_3_ on tuber quality in potato (*Solanum tuberosum* L.). *Agric. Ecosyst. Environ.* 87 273–285. 10.1016/S0167-8809(01)00144-X

[B13] Drake J. E. Gallet-Budynek A. Hofmockel K. S. Bernhardt E. S. Billings S. A. Jackson R. B. (2011). Increases in the flux of carbon belowground stimulate nitrogen uptake and sustain the long-term enhancement of forest productivity under elevated CO_2_. *Ecol. Lett.* 14 349–357. 10.1111/j.1461-0248.2011.01593.x 21303437

[B14] Egger M. Smith G. D. Schneider M. Minder C. (1997). Bias in meta-analysis detected by a simple, graphical test. *BMJ* 315 629–634. 10.1136/bmj.315.7109.629 9310563 PMC2127453

[B15] Fangmeier A. De Temmerman L. Black C. Persson K. Vorne V. (2002). Effects of elevated CO_2_ and/or ozone on nutrient concentrations and nutrient uptake of potatoes. *Eur. J. Agron.* 17 353–368. 10.1016/S1161-0301(02)00071-0

[B16] Fierro A. Gosselin A. Tremblay N. (1994). Supplemental carbon dioxide and light improved tomato and pepper seedling growth and yield. *Hortscience* 29 152–154.

[B17] Fu Y. Shao L. Liu H. Li H. Zhao Z. Ye P. (2015). Unexpected decrease in yield and antioxidants in vegetable at very high CO_2_ levels. *Environ. Chem. Lett.* 13 473–479. 10.1007/s10311-015-0522-6

[B18] Gruda N. (2005). Impact of environmental factors on product quality of greenhouse vegetables for fresh consumption. *Crit. Rev. Plant Sci.* 24 227–247. 10.1080/07352680591008628

[B19] Gruda N. Tanny J. (2014). “Protected Crops,” in *Horticulture: Plants for People and Places: Production Horticulture* Vol. 1 eds Dixon G. R. Aldous D. E. (Dordrecht: Springer), 327–405. 10.1007/978-94-017-8578-5_10

[B20] Gruda N. Tanny J. (2015). *Protected Crops-Recent Advances, Innovative Technologies and Future Challenges*. Leuven: International Society for Horticultural Science (ISHS), 271–278. 10.17660/ActaHortic.2015.1107.37

[B21] Heagle A. S. Miller J. E. Pursley W. A. (2003). Growth and yield responses of potato to mixtures of carbon dioxide and ozone. *J. Environ. Qual.* 32 1603–1610. 10.2134/jeq2003.1603 14535300

[B22] Hedges L. V. Gurevitch J. Curtis Peter S. (1999). The meta-analysis of response ratios in experimental ecology. *Ecology* 80 1150–1156. 10.1890/0012-9658(1999)080[1150:TMAORR]2.0.CO;2

[B23] Helyes L. Lugasi A. Neményi A. Pék Z. (2012). The simultaneous effect of elevated CO2-level and nitrogen-supply on the fruit components of tomato. *Acta Aliment* 41 265–271. 10.1556/AAlim.41.2012.2.13

[B24] Högy P. Fangmeier A. (2009). Atmospheric CO_2_ enrichment affects potatoes: 2. Tuber quality traits. *Eur. J. Agron.* 30 85–94. 10.1016/j.eja.2008.07.006

[B25] Huang B. Xu Y. (2015). Cellular and molecular mechanisms for elevated CO_2_-regulation of plant growth and stress adaptation. *Crop Sci.* 55 1–20. 10.2135/cropsci2014.07.0508

[B26] Idso S. B. Idso K. E. (2001). Effects of atmospheric CO_2_ enrichment on plant constituents related to animal and human health. *Environ. Exp. Bot.* 45 179–199. 10.1016/S0098-8472(00)00091-5 11275225

[B27] IPCC (2014). *Climate Change 2014: Synthesis Report. Contribution of Working Groups I, II and III to the Fifth Assessment Report of the Intergovernmental Panel on Climate Change*, eds Core Writing Team Pachauri R. K. Meyer L. A. Geneva: IPCC, 40–54.

[B28] Islam M. Matsui T. Yoshida Y. (1994). Effects of carbon dioxide enrichment on acid invertase and sugar concentration in developing tomato fruit. *Environ. Control Biol.* 32 245–251. 10.2525/ecb1963.32.245

[B29] Islam S. M. Matsui T. Yoshida Y. (1996). Effect of carbon dioxide enrichment on physico-chemical and enzymatic changes in tomato fruits at various stages of maturity. *Sci. Hortic.* 65 137–149. 10.1016/0304-4238(95)00867-5

[B30] Jaafar H. Z. E. Ibrahim M. H. Karimi E. (2012). Phenolics and flavonoids compounds, phenylanine ammonia lyase and antioxidant activity responses to elevated CO_2_ in *Labisia pumila* (Myrisinaceae). *Molecules* 17 6331–6347. 10.3390/molecules17066331 22634843 PMC6268359

[B31] Jablonski L. M. Wang X. Curtis P. S. (2002). Plant reproduction under elevated CO_2_ conditions: a meta-analysis of reports on 79 crop and wild species. *New Phytol.* 156 9–26. 10.1046/j.1469-8137.2002.00494.x

[B32] Jain V. Pal M. Raj A. Khetarpal S. (2007). Photosynthesis and nutrient composition of spinach and fenugreek grown under elevated carbon dioxide concentration. *Biol. Plant.* 51 559–562. 10.1007/s10535-007-0122-9

[B33] Jin C. Du S. Wang Y. Condon J. Lin X. Zhang Y. (2009). Carbon dioxide enrichment by composting in greenhouses and its effect on vegetable production. *J. Plant Nutr. Soil Sci.* 172 418–424. 10.1002/jpln.200700220

[B34] Khan I. Azam A. Mahmood A. (2013). The impact of enhanced atmospheric carbon dioxide on yield, proximate composition, elemental concentration, fatty acid and vitamin C contents of tomato (*Lycopersicon esculentum*). *Environ. Monit. Assess.* 185 205–214. 10.1007/s10661-012-2544-x 22382378

[B35] Kimball B. A. (1983). Carbon dioxide and agricultural yield: an assemblage and analysis of 430 prior observations. *Agron. J.* 75 779–788. 10.2134/agronj1983.00021962007500050014x

[B36] Kirby K. N. Gerlanc D. (2013). BootES: an R package for bootstrap confidence intervals on effect sizes. *Behav. Res.* 45 905–927. 10.3758/s13428-013-0330-5 23519455

[B37] Kirschbaum M. U. (2011). Does enhanced photosynthesis enhance growth? Lessons learned from CO_2_ enrichment studies. *Plant Physiol.* 155 117–124. 10.1104/pp.110.166819 21088226 PMC3075783

[B38] Krumbein A. Schwarz D. Kläring H. P. (2006). Effects of environmental factors on carotenoid content in tomato (*Lycopersicon esculentum* L. Mill.) grown in a greenhouse. *J. Appl. Bot. Food Qual.* 80 160–164.

[B39] Kumari S. Agrawal M. (2014). Growth, yield and quality attributes of a tropical potato variety (*Solanum tuberosum* L. cv Kufri chandramukhi) under ambient and elevated carbon dioxide and ozone and their interactions. *Ecotoxicol. Environ. Saf.* 101 146–156. 10.1016/j.ecoenv.2013.12.021 24507140

[B40] Kumari S. Agrawal M. Tiwari S. (2013). Impact of elevated CO_2_ and elevated O_3_ on *Beta vulgaris* L.: pigments, metabolites, antioxidants, growth and yield. *Environ. Pollut.* 174 279–288. 10.1016/j.envpol.2012.11.021 23291007

[B41] La G. Fang P. Teng Y. Li Y. Lin X. (2009). Effect of CO_2_ enrichment on the glucosinolate contents under different nitrogen levels in bolting stem of Chinese kale (*Brassica alboglabra* L.). *J. Zhejiang Univ. Sci. B* 10 454–464. 10.1631/jzus.B0820354 19489111 PMC2689558

[B42] Lam S. K. Chen D. Norton R. Armstrong R. Mosier A. R. (2012). Nitrogen dynamics in grain crop and legume pasture systems under elevated atmospheric carbon dioxide concentration: a meta-analysis. *Glob. Change Biol.* 18 2853–2859. 10.1111/j.1365-2486.2012.02758.x 24501062

[B43] Levine L. H. Paré P. W. (2009). Antioxidant capacity reduced in scallions grown under elevated CO_2_ independent of assayed light intensity. *Adv. Space Res.* 44 887–894. 10.1016/j.asr.2009.06.017

[B44] Li F. Wang J. Chen Y. Zou Z. Wang X. Yue M. (2007). Combined effects of enhanced ultraviolet-B radiation and doubled CO_2_ concentration on growth, fruit quality and yield of tomato in winter plastic greenhouse. *Front. Biol. China* 2:414–418. 10.1007/s11515-007-0063-x

[B45] Li J. H. Sagi M. Gale J. Volokita M. Novoplansky A. (1999). Response of tomato plants to saline water as affected by carbon dioxide supplementation. I. Growth, yield and fruit quality. *J. Hortic. Sci. Biotechnol.* 74 232–237. 10.1080/14620316.1999.11511100

[B46] Li X. Kang S. Li F. Zhang X. Huo Z. Ding R. (2017). Light supplement and carbon dioxide enrichment affect yield and quality of off-season pepper. *Agron. J.* 109 2107–2118. 10.2134/agronj2017.01.0044

[B47] Liu J. Feng K. Wang G. Xu Z. Wang F. Xiong A. (2018). Elevated CO_2_ induces alteration in lignin accumulation in celery (*Apium graveolens* L.). *Plant Physiol. Biochem.* 127 310–319. 10.1016/j.plaphy.2018.04.003 29653434

[B48] Loladze I. (2014). Hidden shift of the ionome of plants exposed to elevated CO_2_ depletes minerals at the base of human nutrition. *eLife* 3:e02245. 10.7554/eLife.02245 24867639 PMC4034684

[B49] Long S. P. Ainsworth E. A. Rogers A. Ort D. R. (2004). Rising atmospheric carbon dioxide: plants FACE the Future. *Annu. Rev. Plant Biol.* 55 591–628. 10.1146/annurev.arplant.55.031903.141610 15377233

[B50] Mamatha H. Srinivasa Rao N. K. Laxman R. H. Shivashankara K. S. Bhatt R. M. Pavithra K. C. (2014). Impact of elevated CO_2_ on growth, physiology, yield, and quality of tomato (*Lycopersicon esculentum* Mill) cv. Arka Ashish. *Photosynthetica* 52 519–528. 10.1007/s11099-014-0059-0

[B51] McDonald E. P. Erickson J. E. Kruger E. L. (2002). Can decreased transpiration limit plant nitrogen acquisition in elevated CO_2_? *Funct. Plant Biol.* 29 1115–1120. 10.1071/FP02007 32689563

[B52] Miyagi A. Uchimiya H. Kawai-Yamada M. (2017). Synergistic effects of light quality, carbon dioxide and nutrients on metabolite compositions of head lettuce under artificial growth conditions mimicking a plant factory. *Food Chem.* 218 561–568. 10.1016/j.foodchem.2016.09.102 27719950

[B53] Mizrahi Y. Pasternak D. O. V. (1985). Effect of salinity on quality of various agricultural crops. *Plant Soil* 89 301–307. 10.1007/BF02182249

[B54] Moretti C. L. Mattos L. M. Calbo A. G. Sargent S. A. (2010). Climate changes and potential impacts on postharvest quality of fruit and vegetable crops: a review. *Food Res. Int.* 43 1824–1832. 10.1016/j.foodres.2009.10.013

[B55] Mortensen L. M. (1987). Review: CO_2_ enrichment in greenhouses. Crop responses. *Sci. Hortic.* 33 1–25. 10.1016/j.scitotenv.2016.07.030 27424117

[B56] Mortensen L. M. (1994). Effects of elevated CO_2_ concentrations on growth and yield of eight vegetable species in a cool climate. *Sci. Hortic.* 58 177–185. 10.1016/0304-4238(94)90149-X

[B57] Myers S. S. Zanobetti A. Kloog I. Huybers P. Leakey A. D. B. Bloom A. J. (2014). Increasing CO_2_ threatens human nutrition. *Nature* 510 139–142. 10.1038/nature13179 24805231 PMC4810679

[B58] Nitithamyong A. Vonelbe J. H. Wheeler R. M. Tibbitts T. W. (1999). Glycoalkaloids in potato tubers grown under controlled environments. *Am. J. Potato Res.* 76 337–343. 10.1007/BF02910006 11543354

[B59] Pérez-López U. Miranda-Apodaca J. Lacuesta M. Mena-Petite A. Muñoz-Rueda A. (2015a). Growth and nutritional quality improvement in two differently pigmented lettuce cultivars grown under elevated CO_2_ and/or salinity. *Sci. Hortic.* 195 56–66. 10.1016/j.scienta.2015.08.034

[B60] Pérez-López U. Miranda-Apodaca J. Muñoz-Rueda A. Mena-Petite A. (2013). Lettuce production and antioxidant capacity are differentially modified by salt stress and light intensity under ambient and elevated CO_2_. *J. Plant Physiol.* 170 1517–1525. 10.1016/j.jplph.2013.06.004 23838124

[B61] Pérez-López U. Miranda-Apodaca J. Muñoz-Rueda A. Mena-Petite A. (2015b). Interacting effects of high light and elevated CO_2_ on the nutraceutical quality of two differently pigmented *Lactuca sativa* cultivars (Blonde of Paris Batavia and Oak Leaf). *Sci. Hortic.* 191 38–48. 10.1016/j.scienta.2015.04.030

[B62] Pérez-López U. Sgherri C. Miranda-Apodaca J. Micaelli F. Lacuesta M. Mena-Petite A. (2018). Concentration of phenolic compounds is increased in lettuce grown under high light intensity and elevated CO_2_. *Plant Physiol. Biochem.* 123 233–241. 10.1016/j.plaphy.2017.12.010 29253801

[B63] Piñero M. C. Pérez-Jiménez M. López-Marín J. del Amor F. M. (2017a). Fruit quality of sweet pepper as affected by foliar Ca applications to mitigate the supply of saline water under a climate change scenario. *J. Sci. Food Agric.* 98 1071–1078. 10.1002/jsfa.8557 28722753

[B64] Piñero M. C. Otálora G. Porras M. E. Sánchez-Guerrero M. C. Lorenzo P. Medrano E. (2017b). The form in which nitrogen is supplied affects the polyamines, amino acids, and mineral composition of sweet pepper fruit under an elevated CO_2_ concentration. *J. Agric. Food Chem.* 65 711–717. 10.1021/acs.jafc.6b04118 28075582

[B65] Proietti S. Moscatello S. Giacomelli G. A. Battistelli A. (2013). Influence of the interaction between light intensity and CO_2_ concentration on productivity and quality of spinach (*Spinacia oleracea* L.) grown in fully controlled environment. *Adv. Space Res.* 52 1193–1200. 10.1016/j.asr.2013.06.005

[B66] Rao M. V. Hale B. A. Ormrod D. P. (1995). Amelioration of ozone-induced oxidative damage in wheat plants grown under high carbon dioxide (role of antioxidant enzymes). *Plant Physiol.* 109 421–432. 10.1104/pp.109.2.421 12228603 PMC157604

[B67] Reich M. van den Meerakker A. N. Parmar S. Hawkesford M. J. De Kok L. J. (2016). Temperature determines size and direction of effects of elevated CO_2_ and nitrogen form on yield quantity and quality of Chinese cabbage. *Plant Biol.* 18 63–75. 10.1111/plb.12396 26390257

[B68] Ren J. Guo S.-S. Xin X.-L. Chen L. (2014). Changes in volatile constituents and phenols from *Gynura bicolor* DC grown in elevated CO_2_ and LED lighting. *Sci. Hortic.* 175 243–250. 10.1016/j.scienta.2014.06.023

[B69] Robredo A. Pérez-López U. Miranda-Apodaca J. Lacuesta M. Mena-Petite A. Muñoz-Rueda A. (2011). Elevated CO_2_ reduces the drought effect on nitrogen metabolism in barley plants during drought and subsequent recovery. *Environ. Exp. Bot.* 71 399–408. 10.1016/j.envexpbot.2011.02.011

[B70] Roosta H. R. Schjoerring J. K. (2008). Effects of nitrate and potassium on ammonium toxicity in cucumber plants. *J. Plant Nutr.* 31 1270–1283. 10.1080/01904160802135050

[B71] Schjoerring J. K. Husted S. Mäck G. Mattsson M. (2002). The regulation of ammonium translocation in plants. *J. Exp. Bot.* 53 883–890. 10.1093/jexbot/53.370.883 11912231

[B72] Sgherri C. Pérez-López U. Micaelli F. Miranda-Apodaca J. Mena-Petite A. Muñoz-Rueda A. (2017). Elevated CO_2_ and salinity are responsible for phenolics-enrichment in two differently pigmented lettuces. *Plant Physiol. Biochem.* 115 269–278. 10.1016/j.plaphy.2017.04.006 28411511

[B73] Stitt M. Krapp A. (1999). The interaction between elevated carbon dioxide and nitrogen nutrition: the physiological and molecular background. *Plant Cell Environ.* 22 583–621. 10.1046/j.1365-3040.1999.00386.x

[B74] Sun P. Mantri N. Lou H. Hu Y. Sun D. Zhu Y. (2012). Effects of elevated CO_2_ and temperature on yield and fruit quality of strawberry (*Fragaria × ananassa* Duch.) at two levels of nitrogen application. *PLoS One* 7:e41000. 10.1371/journal.pone.0041000 22911728 PMC3404062

[B75] Tang Y. Dong J. Li X. Gruda N. Duan Z. (2018). Interactive effects of elevated carbon dioxide and nitrogen availability on fruit quality of cucumber (*Cucumis sativus* L.). *J. Integr. Agric.* 29479715 10.1002/jsfa.8976

[B76] Taub D. R. Miller B. Allen H. (2008). Effects of elevated CO_2_ on the protein concentration of food crops: a meta-analysis. *Glob. Change Biol.* 14 565–575. 10.1111/j.1365-2486.2007.01511.x

[B77] Thompson L. Peffley E. Green C. Pare P. Tissue D. (2004). “Biomass, flavonol levels and sensory characteristics of Allium cultivars grown hydroponically at ambient and elevated CO_2_,” in *Proceedings of the 34th International Conferences on Environmental Systems (ICES)* Colorado Springs, Colorado 10.4271/2004-01-2300

[B78] van Groenigen K. J. Osenberg C. W. Hungate B. A. (2011). Increased soil emissions of potent greenhouse gases under increased atmospheric CO_2_. *Nature* 475 214–218. 10.1038/nature10176 21753852

[B79] Vorne V. Ojanperä K. De Temmerman L. Bindi M. Högy P. Jones M. (2002). Effects of elevated carbon dioxide and ozone on potato tuber quality in the European multiple-site experiment ‘CHIP-project’. *Eur. J. Agron.* 17 369–381. 10.1016/S1161-0301(02)00072-2

[B80] Wang D. Heckathorn S. A. Wang X. Philpott S. M. (2012). A meta-analysis of plant physiological and growth responses to temperature and elevated CO_2_. *Oecologia* 169 1–13. 10.1007/s00442-011-2172-0 22037993

[B81] Wang S. Y. Bunce J. A. (2004). Elevated carbon dioxide affects fruit flavor in field-grown strawberries (*Fragaria × ananassa* Duch). *J. Sci. Food Agric.* 84 1464–1468. 10.1002/jsfa.1824

[B82] Wang S. Y. Bunce J. A. Maas J. L. (2003). Elevated carbon dioxide increases contents of antioxidant compounds in field-grown strawberries. *J. Agric. Food Chem.* 51 4315–4320. 10.1021/jf021172d 12848504

[B83] Wei Z. Du T. Li X. Fang L. Liu F. (2018). Interactive effects of elevated CO_2_ and N fertilization on yield and quality of tomato grown under reduced irrigation regime. *Front. Plant Sci.* 9:328. 10.3389/fpls.2018.00328 29636756 PMC5880949

[B84] Winsor G. W. Davies J. N. Massey D. M. (1962). Composition of tomato fruit. IV.—Changes in some constituents of the fruit walls during ripening. *J. Sci. Food Agric.* 13 141–145. 10.1002/jsfa.2740130301 22623518

[B85] Wu X. Sun S. Xing G. Wang G. Wang F. Xu Z. (2017). Elevated carbon dioxide altered morphological and anatomical characteristics, ascorbic acid accumulation, and related gene expression during taproot development in carrots. *Front. Plant Sci.* 7:2026. 10.3389/fpls.2016.02026 28119712 PMC5221676

[B86] Xu Z. Jiang Y. Zhou G. (2015). Response and adaptation of photosynthesis, respiration, and antioxidant systems to elevated CO_2_ with environmental stress in plants. *Front. Plant Sci.* 6:701. 10.3389/fpls.2015.00701 26442017 PMC4564695

[B87] Zhang Z. Liu L. Zhang M. Zhang Y. Wang Q. (2014). Effect of carbon dioxide enrichment on health-promoting compounds and organoleptic properties of tomato fruits grown in greenhouse. *Food Chem.* 153 157–163. 10.1016/j.foodchem.2013.12.052 24491715

