# Supplementary material for: Effects of Elevated CO2 on Nutritional Quality of Vegetables: A Review
Source: Front Plant Sci. 2018 Aug 15;9:924. doi: 10.3389/fpls.2018.00924 (PMC6104417; doi:10.3389/fpls.2018.00924)
Supplement: Supplementary file 1 [file Data_Sheet_1.docx]

**Supplementary information**

**Effects of Elevated CO_2_ on Nutritional Quality of Vegetables – A Review**

By Dong et al

**Table S1. Effects of elevated CO_2_ on concentrations of carbohydrates in vegetables.**

| Quality parameters | CO_2_ effects | Crops |
| --- | --- | --- |
| Fructose | ↑ | ^‡^Broccoli (Krumbein et al., 2010), ^‡§^cucumber (Dong et al., 2018; Tang et al., 2018),^§^lettuce (Becker and Kläring, 2016), ^†‡^potato (Högy and Fangmeier, 2009), ^‡§^strawberry (Sun et al., 2012; Wang and Bunce, 2004) and ^§^tomato (Behboudian and Tod, 1995; Islam et al., 1994; Islam et al., 1996) |
|  | = | ^‡§^Cucumber (Dong et al., 2018; Tang et al., 2018), ^†‡^potato (Donnelly et al., 2001; Vorne et al., 2002) |
| Glucose | ↑ | ^‡§^Cucumber (Dong et al., 2018; Tang et al., 2018), ^§^lettuce (Becker and Kläring, 2016), ^†‡^potato (Högy and Fangmeier, 2009), ^‡§^strawberry (Sun et al., 2012; Wang and Bunce, 2004) and ^§^tomato (Behboudian and Tod, 1995; Islam et al., 1994; Islam et al., 1996) |
|  | = | ^‡^Broccoli (Krumbein et al., 2010), ^‡§^cucumber (Dong et al., 2018; Tang et al., 2018), and ^†‡^potato (Donnelly et al., 2001; Vorne et al., 2002) |
| Sucrose | ↑ | ^§^Lettuce (Becker and Kläring, 2016), ^§^spinach (Proietti et al., 2013), ^‡§^strawberry (Wang and Bunce, 2004) and ^§^tomato (Islam et al., 1996) |
|  | = | ^‡^Broccoli (Krumbein et al., 2010), ^†‡^potato (Donnelly et al., 2001; Högy and Fangmeier, 2009; Vorne et al., 2002), ^‡^strawberry (Sun et al., 2012), ^†‡^sugar beet (Demmers-Derks et al., 1998; Manderscheid et al., 2010) and ^§^tomato (Behboudian and Tod, 1995; Islam et al., 1994; Islam et al., 1996) |
| Total soluble sugar | ↑ | ^§^Chinese cabbage, lettuce and oily sowthistle (Jin et al., 2009), ^‡§^lettuce (Baslam et al., 2012; Becker and Kläring, 2016; Pérez-López et al., 2015a), ^‡^onion (Bettoni et al., 2017), ^†^palak (Kumari et al., 2013), ^†^potato (Kumari and Agrawal, 2014), ^§^radish and turnip (Azam et al., 2013), ^§^spinach (Proietti et al., 2013), ^‡§^strawberry (Sun et al., 2012; Wang and Bunce, 2004) and ^‡§^tomato (Behboudian and Tod, 1995; Helyes et al., 2012; Islam et al., 1994; Khan et al., 2013; Li et al., 2007; Zhang et al., 2014) |
|  | = | ^‡^Broccoli (Krumbein et al., 2010), ^§^celtuce (Jin et al., 2009), ^‡^hot pepper(Li et al., 2017), ^‡§^lettuce (Baslam et al., 2012; Jin et al., 2009; Pérez-López et al., 2015a; Pérez-López et al., 2015b), ^†‡^potato (Donnelly et al., 2001; Vorne et al., 2002) and ^‡§^tomato (Li et al., 1999; Wei et al., 2018) |
|  | ↓ | ^§^Celery (Jin et al., 2009), ^†‡§^hot pepper (Li et al., 2017) and ^‡^tomato (Helyes et al., 2012) |
| Reducing sugar | ↑ | ^†^Palak (Kumari et al., 2013), ^†‡^potato (Högy and Fangmeier, 2009), ^§^radish and turnip (Azam et al., 2013) and ^§^tomato (Islam et al., 1996; Khan et al., 2013) |
|  | ↓ | ^†^Potato (Kumari and Agrawal, 2014) |
| Non-reducing sugar | ↑ | ^§^Radish (Azam et al., 2013) |
|  | = | ^§^Tomato (Khan et al., 2013) and ^§^turnip (Azam et al., 2013) |
| Starch | ↑ | ^‡§^Cucumber (Dong et al., 2018), ^‡^lettuce (Pérez-López et al., 2015b), ^‡^onion (Bettoni et al., 2017), ^†^palak (Kumari et al., 2013) and ^†‡^potato (Donnelly et al., 2001; Kumari and Agrawal, 2014; Vorne et al., 2002) |
|  | = | ^‡§^Cucumber (Dong et al., 2018; Tang et al., 2018), ^‡^lettuce (Baslam et al., 2012; Pérez-López et al., 2015a; Pérez-López et al., 2015b) , ^‡^onion (Bettoni et al., 2017) and ^†‡^potato (Donnelly et al., 2001; Högy and Fangmeier, 2009; Vorne et al., 2002) |
| Lignin | ↑/= | ^§^Celery (Liu et al., 2018) |
| Dietary fibre | ↑ | ^§^Carrot, radish and turnip (Azam et al., 2013), ^‡§^cucumber (Dong et al., 2018) and ^§^tomato (Khan et al., 2013) |
|  | = | ^‡^Cucumber (Dong et al., 2018; Tang et al., 2018) and ^§^tomato (Khan et al., 2013) |
|  | ↓ | ^‡§^Cucumber (Tang et al., 2018) |

↑ increase, ＝ no significant change, ↓ decrease (*p*<0.05). †, ‡ and § indicate the ratios of concentrations of elevated CO_2_ to ambient CO_2_ are <1.5, 1.5-2.0 and >2.0, respectively.

**Table S2. Effects of elevated CO_2_ on concentrations of taste-related organic acids in vegetables.**

| Quality parameters | CO_2_ effects | Crops |
| --- | --- | --- |
| Malic acid | ↑ | ^§^Lettuce (Miyagi et al., 2017) |
|  | = | ^§^Lettuce (Miyagi et al., 2017), ^†‡^potato (Donnelly et al., 2001; Högy and Fangmeier, 2009; Vorne et al., 2002) and ^‡^strawberry (Wang and Bunce, 2004) |
|  | ↓ | ^§^Strawberry (Wang and Bunce, 2004) and ^§^tomato (Islam et al., 1996) |
| Citric acid | ↑ | ^§^Lettuce (Miyagi et al., 2017) |
|  | = | ^‡^Potato (Donnelly et al., 2001) and ^‡^strawberry (Wang and Bunce, 2004) |
|  | ↓ | ^†‡^Potato (Donnelly et al., 2001; Högy and Fangmeier, 2009; Vorne et al., 2002), ^§^strawberry (Wang and Bunce, 2004) and ^§^tomato (Islam et al., 1996) |
| Oxalic acid | ↑/= | ^§^Spinach (Proietti et al., 2013) |
|  | ↓ | ^§^Lettuce (Miyagi et al., 2017) and ^§^tomato (Islam et al., 1996) |
| Titratable acidity | ↑ | ^‡^Onion (Bettoni et al., 2017) and ^§^tomato (Zhang et al., 2014) |
|  | = | ^§^Carrot, radish and turnip (Azam et al., 2013), ^‡§^cucumber (Tang et al., 2018), ^‡^onion (Bettoni et al., 2017) and ^§^tomato (Khan et al., 2013; Li et al., 1999; Özçelik and Akilli, 1999; Zhang et al., 2014) |
|  | ↓ | ^‡^Onion (Bettoni et al., 2017) and ^†‡^tomato (Mamatha et al., 2014) |
| Sugar/acid | ↑ | ^‡§^Cucumber (Tang et al., 2018), ^§^strawberry (Wang and Bunce, 2004) and ^‡§^tomato (Wei et al., 2018; Zhang et al., 2014) |
|  | = | ^‡§^Cucumber (Tang et al., 2018) and ^‡§^tomato (Wei et al., 2018; Zhang et al., 2014) |
|  | ↓ | ^‡^Tomato (Wei et al., 2018) |

↑ increase, ＝ no significant change, ↓ decrease (*p*<0.05). †, ‡ and § indicate the ratios of concentrations of elevated CO_2_ to ambient CO_2_ are <1.5, 1.5-2.0 and >2.0, respectively.

**Table S3. Effects of elevated CO_2_ on concentrations of several nitrogenous compounds in vegetables.**

| Quality parameters | CO_2_ effects | Crops |
| --- | --- | --- |
| Total protein | ↑ | ^‡^Fenugreek (Jain et al., 2007), ^‡^lettuce (Baslam et al., 2012), ^†^palak (Kumari et al., 2013) and ^†^potato (Kumari and Agrawal, 2014) |
|  | = | ^‡^Cabbage (Reddy et al., 2004), ^‡§^cucumber (Dong et al., 2018; Tang et al., 2018) and ^‡^lettuce (Baslam et al., 2012; Pérez-López et al., 2015a; Pérez-López et al., 2015b), ^‡^spinach (Jain et al., 2007), ^‡^strawberry (Sun et al., 2012) and ^§^sweet pepper (Piñero et al., 2017b) |
|  | ↓ | ^‡^Broccoli (Schonhof et al., 2007), ^§^carrot, radish and turnip (Azam et al., 2013), ^§^Chines kale (La et al., 2009), ^‡§^cucumber (Dong et al., 2018; Tang et al., 2018), ^‡^fenugreek and spinach (Jain et al., 2007), ^‡^lettuce (Baslam et al., 2012; Pérez-López et al., 2015a), ^†‡^potato (Donnelly et al., 2001; Fangmeier et al., 2002; Heagle et al., 2003; Högy and Fangmeier, 2009; Kumari and Agrawal, 2014), ^†^palak (Kumari et al., 2013), sweet pepper (Piñero et al., 2017a; Piñero et al., 2017b) and ^§^tomato (Behboudian and Tod, 1995; Khan et al., 2013) |
| Soluble protein | ↑ | ^‡^Onion (Bettoni et al., 2017),^†^palak (Kumari et al., 2013) and ^†^potato (Kumari and Agrawal, 2014) |
|  | = | ^†‡§^Hot pepper (Li et al., 2017) and ^‡^lettuce (Pérez-López et al., 2015a; Pérez-López et al., 2015b) |
|  | ↓ | ^‡§^Hot pepper (Li et al., 2017), ^‡^lettuce (Baslam et al., 2012), ^†^palak (Kumari et al., 2013) and ^†^potato (Kumari and Agrawal, 2014) |
| ⍺-amino-nitrogen | = | ^†‡^Sugar beet (Demmers-Derks et al., 1998; Manderscheid et al., 2010) |
|  | ↓ | ^‡^Sugar beet (Demmers-Derks et al., 1998) |
| Free amino acids | ↑ | ^§^Lettuce (Miyagi et al., 2017) and ^†^potato (Kumari and Agrawal, 2014) |
|  | = | ^‡^Chinese cabbage (Reich et al., 2016) and ^§^lettuce (Miyagi et al., 2017) |
|  | ↓ | ^†^Potato (Kumari and Agrawal, 2014) |
| Nitrate | ↑ | ^§^Celtuce (Jin et al., 2009), ^‡^lettuce (Pérez-López et al., 2015a; Pérez-López et al., 2015b) and ^‡^tomato (Wei et al., 2018) |
|  | = | ^‡^Chinese cabbage (Reich et al., 2016), ^‡§^cucumber (Dong et al., 2018; Tang et al., 2018), ^‡^lettuce (Pérez-López et al., 2015a; Pérez-López et al., 2015b), ^†‡^potato (Donnelly et al., 2001; Högy and Fangmeier, 2009), ^§^spinach (Proietti et al., 2013) and ^‡§^tomato (Wei et al., 2018; Wheeler et al., 1997) |
|  | ↓ | ^§^Celery, Chinese cabbage, lettuce and oily sowthistle (Jin et al., 2009), ^‡§^cucumber (Dong et al., 2018; Tang et al., 2018), ^‡^lettuce (Pérez-López et al., 2015a), ^†‡^potato (Donnelly et al., 2001; Vorne et al., 2002) and ^§^spinach (Proietti et al., 2013) |

↑ increase, ＝ no significant change, ↓ decrease (*p*<0.05). †, ‡ and § indicate the ratios of concentrations of elevated CO_2_ to ambient CO_2_ are <1.5, 1.5-2.0 and >2.0, respectively.

**Table S4. Effects of elevated CO_2_ on concentrations of antioxidants in vegetables.**

| Quality parameters | CO_2_ effects | Crops |
| --- | --- | --- |
| Ascorbic acid | ↑ | ^§^Celery, celtuce, Chinese cabbage, lettuce and oily sowthistle (Jin et al., 2009), ^§^Chinese cabbage and lettuce (Fu et al., 2015), ^‡^lettuce (Baslam et al., 2012), ^‡^potato (Donnelly et al., 2001), ^§^spinach (Proietti et al., 2013), ^‡§^strawberry (Wang et al., 2003) and ^†‡§^tomato (Islam et al., 1996; Li et al., 2007; Mamatha et al., 2014; Zhang et al., 2014) |
|  | = | ^†‡§^Hot pepper (Li et al., 2017), ^‡^lettuce (Baslam et al., 2012; Pérez-López et al., 2015a; Pérez-López et al., 2015b), ^†^palak (Kumari et al., 2013), ^†‡^potato (Högy and Fangmeier, 2009; Vorne et al., 2002), ^§^spinach (Seo et al., 2017) and ^§^tomato(Özçelik and Akilli, 1999) |
|  | ↓ | ^§^Carrot, radish and turnip (Azam et al., 2013), ^†‡§^hot pepper (Li et al., 2017) and ^§^tomato (Khan et al., 2013) |
| Total flavonoids | ↑ | ^§^Chinese cabbage and lettuce (Fu et al., 2015), ^‡^ginger (Ghasemzadeh et al., 2010), ^§^hongfengcai (Ren et al., 2014), ^‡§^lettuce (Becker and Kläring, 2016; Pérez-López et al., 2018; Sgherri et al., 2017), ^§^onion and scallion (Thompson et al., 2004) and ^§^strawberry (Wang et al., 2003) |
|  | = | ^‡§^Lettuce (Becker and Kläring, 2016; Pérez-López et al., 2018) and ^§^chives and scallion (Thompson et al., 2004) |
|  | ↓ | ^‡§^Strawberry (Sun et al., 2012) and ^†‡^tomato (Mamatha et al., 2014) |
| Anthocyanins | ↑ | ^§^Hongfengcai (Ren et al., 2014), ^‡§^lettuce (Baslam et al., 2012; Becker and Kläring, 2016) and ^§^strawberry (Wang et al., 2003) |
|  | = | ^‡^Lettuce (Baslam et al., 2012) |
|  | ↓ | ^‡^Lettuce (Baslam et al., 2012; Pérez-López et al., 2015a; Pérez-López et al., 2015b) and ^‡^strawberry (Sun et al., 2012) |
| Total phenols | ↑ | ^§^Chinese cabbage (Fu et al., 2015), ^‡^ginger (Ghasemzadeh et al., 2010), ^§^hongfengcai (Ren et al., 2014), ^‡§^lettuce (Baslam et al., 2012; Fu et al., 2015; Pérez-López et al., 2015a; Pérez-López et al., 2018; Sgherri et al., 2017), ^‡^onion (Bettoni et al., 2017) and ^‡^tomato (Helyes et al., 2012) |
|  | = | ^‡^Cabbage (Reddy et al., 2004), ^§^hongfengcai (Ren et al., 2014), ^‡^lettuce (Baslam et al., 2012; Pérez-López et al., 2015a), ^†^palak (Kumari et al., 2013), ^§^scallion (Levine and Paré, 2009) and ^§^sweet pepper (Piñero et al., 2017b) |
|  | ↓ | ^‡^Lettuce (Pérez-López et al., 2015b; Pérez-López et al., 2018), ^‡^strawberry (Sun et al., 2012) and ^†‡^tomato (Helyes et al., 2012; Mamatha et al., 2014) |
| Glutathione | ↑ | ^§^Hongfengcai (Wang et al., 2016), ^‡^lettuce (Pérez-López et al., 2015a) and ^‡§^strawberry (Wang et al., 2003) |
|  | = | ^§^Hongfengcai (Wang et al., 2016) and ^‡^lettuce (Pérez-López et al., 2015a; Pérez-López et al., 2015b) |
| Lycopene | ↑ | ^‡§^Tomato (Helyes et al., 2012; Zhang et al., 2014) |
|  | = | ^§^Sweet pepper (Piñero et al., 2017b) and ^†‡§^tomato (Helyes et al., 2012; Krumbein et al., 2006; Li et al., 2007; Mamatha et al., 2014; Zhang et al., 2014) |
|  | ↓ | ^‡^Tomato (Helyes et al., 2012; Mamatha et al., 2014) |
| Carotenoids | ↑ | ^§^Hongfengcai (Wang et al., 2016), ^†^palak (Kumari et al., 2013) and ^§^tomato (Zhang et al., 2014) |
|  | = | ^‡^Lettuce (Baslam et al., 2012; Pérez-López et al., 2015a; Pérez-López et al., 2015b), ^†^palak (Kumari et al., 2013) and ^†§^tomato (Mamatha et al., 2014; Zhang et al., 2014) |
|  | ↓ | ^‡^Lettuce (Baslam et al., 2012; Pérez-López et al., 2015b) and ^‡^tomato (Mamatha et al., 2014) |
| Lutein | ↑/= | ^§^Tomato (Zhang et al., 2014) |
| β-carotene | ↑ | ^§^Tomato (Zhang et al., 2014) |
|  | = | ^§^Sweet pepper (Piñero et al., 2017b) and ^§^tomato (Krumbein et al., 2006) |
| Chlorophyll a | ↑ | ^§^Hongfengcai (Wang et al., 2016) |
|  | = | ^‡^Lettuce (Pérez-López et al., 2015a) and ^§^sweet pepper (Piñero et al., 2017b) |
|  | ↓ | ^‡^Lettuce (Pérez-López et al., 2015a; Pérez-López et al., 2015b) |
| Chlorophyll b | ↑ | ^§^Hongfengcai (Wang et al., 2016), ^‡^lettuce (Pérez-López et al., 2015a; Pérez-López et al., 2015b) and ^§^sweet pepper (Piñero et al., 2017b) |
|  | = | ^§^Hongfengcai (Wang et al., 2016) and ^§^sweet pepper (Piñero et al., 2017b) |
| Total chlorophyll | ↑ | ^§^Hongfengcai (Wang et al., 2016) and ^†^palak (Kumari et al., 2013) |
|  | = | ^‡^Lettuce (Baslam et al., 2012) |
|  | ↓ | ^‡^Lettuce (Baslam et al., 2012) |
| Total antioxidant capacity | ↑ | ^§^Chinese cabbage (Fu et al., 2015), ^‡^ginger (Ghasemzadeh et al., 2010), ^§^hongfengcai (Ren et al., 2014; Wang et al., 2016), ^‡§^lettuce (Fu et al., 2015; Pérez-López et al., 2015a; Pérez-López et al., 2013; Pérez-López et al., 2015b; Pérez-López et al., 2018; Sgherri et al., 2017) and ^‡^tomato (Helyes et al., 2012) |
|  | = | ^§^Hongfengcai (Wang et al., 2016) , ^‡^lettuce (Pérez-López et al., 2018), ^§^scallion (Levine and Paré, 2009) and ^‡^tomato (Helyes et al., 2012) |
|  | ↓ | ^‡^Lettuce (Pérez-López et al., 2018), ^§^scallion (Levine and Paré, 2009), ^‡^strawberry (Sun et al., 2012) and ^†‡^tomato (Mamatha et al., 2014) |

↑ increase, ＝ no significant change, ↓ decrease (*p*<0.05). †, ‡ and § indicate the ratios of concentrations of elevated CO_2_ to ambient CO_2_ are <1.5, 1.5-2.0 and >2.0, respectively.

**Table S5. Effects of elevated CO_2_ on concentrations of minerals in vegetables.**

| Quality  parameters | CO_2_  effects | Crops |
| --- | --- | --- |
| P | ↑ | ^‡^Chinese cabbage (Reich et al., 2016), ^§^cucumber (Tang et al., 2018), ^‡^lettuce (Baslam et al., 2012) and ^§^sweet pepper (Piñero et al., 2017b) |
|  | = | ^‡^Chinese cabbage (Reich et al., 2016), ^‡§^cucumber (Dong et al., 2018; Tang et al., 2018), ^‡^lettuce (Baslam et al., 2012; Pérez-López et al., 2015a; Pérez-López et al., 2015b), ^†‡^potato (Fangmeier et al., 2002; Högy and Fangmeier, 2009), ^‡§^sweet pepper (Piñero et al., 2017a; Piñero et al., 2017b) and ^§^tomato (Wheeler et al., 1997) |
|  | ↓ | ^§^Cucumber (Dong et al., 2018), ^‡^lettuce (Baslam et al., 2012; Pérez-López et al., 2015a), ^†‡^potato (Heagle et al., 2003) and ^§^tomato (Behboudian and Tod, 1995) |
| K | ↑ | ^‡^Chinese cabbage (Reich et al., 2016), ^‡§^cucumber (Dong et al., 2018; Tang et al., 2018), ^‡^lettuce (Baslam et al., 2012; Pérez-López et al., 2015a; Pérez-López et al., 2015b), ^†^potato (Kumari and Agrawal, 2014) and ^§^turnip (Azam et al., 2013) |
|  | = | ^§^Carrot and radish (Azam et al., 2013), ^‡^Chinese cabbage (Reich et al., 2016), ^‡§^cucumber (Dong et al., 2018; Tang et al., 2018), ^‡^lettuce (Baslam et al., 2012; Pérez-López et al., 2015a; Pérez-López et al., 2015b), ^†‡^potato (Heagle et al., 2003), ^†^sugar beet (Manderscheid et al., 2010), ^‡§^sweet pepper (Piñero et al., 2017a; Piñero et al., 2017b) and ^§^tomato (Khan et al., 2013; Wheeler et al., 1997) |
|  | ↓ | ^§^Cucumber (Dong et al., 2018), ^‡^lettuce (Baslam et al., 2012; Pérez-López et al., 2015a), ^†‡^potato (Fangmeier et al., 2002; Högy and Fangmeier, 2009; Kumari and Agrawal, 2014), ^§^sweet pepper (Piñero et al., 2017b) and ^§^tomato (Behboudian and Tod, 1995) |
| Ca | ↑ | ^§^Carrot (Azam et al., 2013), ^‡§^cucumber (Dong et al., 2018; Tang et al., 2018), ^‡^fenugreek and spinach (Jain et al., 2007), ^‡^lettuce (Baslam et al., 2012; Pérez-López et al., 2015b), ^†^potato (Kumari and Agrawal, 2014), ^§^sweet pepper (Piñero et al., 2017b) and ^§^tomato (Khan et al., 2013; Wheeler et al., 1997) |
|  | = | ^‡^Chinese cabbage (Reich et al., 2016), ^‡§^cucumber (Dong et al., 2018; Tang et al., 2018), ^‡^lettuce (Pérez-López et al., 2015a; Pérez-López et al., 2015b), ^†‡^potato (Heagle et al., 2003; Högy and Fangmeier, 2009), ^‡^spinach (Jain et al., 2007), ^‡§^sweet pepper (Piñero et al., 2017a; Piñero et al., 2017b) and ^§^tomato (Behboudian and Tod, 1995) |
|  | ↓ | ^‡^Chinese cabbage (Reich et al., 2016), ^§^cucumber (Dong et al., 2018), ^‡^lettuce (Baslam et al., 2012; Pérez-López et al., 2015a), ^†‡^potato (Fangmeier et al., 2002; Kumari and Agrawal, 2014), ^§^radish and turnip (Azam et al., 2013) and ^‡^sweet pepper (Piñero et al., 2017a) |
| Mg | ↑ | ^‡§^Cucumber (Dong et al., 2018; Tang et al., 2018), ^‡^lettuce (Baslam et al., 2012), ^†^potato (Kumari and Agrawal, 2014) and ^§^sweet pepper (Piñero et al., 2017b) |
|  | = | ^§^Carrot (Azam et al., 2013), ^‡^Chinese cabbage (Reich et al., 2016), ^§^cucumber (Tang et al., 2018),  ^‡^lettuce (Baslam et al., 2012; Pérez-López et al., 2015a; Pérez-López et al., 2015b), ^†‡^potato (Fangmeier et al., 2002; Heagle et al., 2003; Högy and Fangmeier, 2009) and ^§^tomato (Behboudian and Tod, 1995; Wheeler et al., 1997) |
|  | ↓ | ^§^Cucumber (Dong et al., 2018), ^‡^fenugreek and spinach (Jain et al., 2007), ^‡^lettuce (Baslam et al., 2012; Pérez-López et al., 2015a; Pérez-López et al., 2015b), ^§^radish and turnip (Azam et al., 2013), ^§^sweet pepper (Piñero et al., 2017b) and ^§^tomato (Khan et al., 2013) |
| S | = | ^‡^Broccoli (Schonhof et al., 2007), ^§^carrot and turnip (Azam et al., 2013), ^‡^Chinese cabbage (Reich et al., 2016), ^‡§^cucumber (Dong et al., 2018; Tang et al., 2018), ^†^potato (Högy and Fangmeier, 2009) and ^§^tomato (Behboudian and Tod, 1995; Khan et al., 2013) |
|  | ↓ | ^‡^Chinese cabbage (Reich et al., 2016), ^§^Chines kale (La et al., 2009) and ^§^cucumber (Dong et al., 2018) |
| Fe | ↑ | ^‡^Lettuce (Baslam et al., 2012), ^†^potato (Kumari and Agrawal, 2014), ^§^radish (Azam et al., 2013) and ^§^tomato (Khan et al., 2013) |
|  | = | ^‡^Chinese cabbage (Reich et al., 2016), ^‡§^cucumber (Dong et al., 2018; Tang et al., 2018), ^‡^fenugreek (Jain et al., 2007), ^‡^lettuce (Baslam et al., 2012; Pérez-López et al., 2015a; Pérez-López et al., 2015b) and ^†‡^potato (Fangmeier et al., 2002; Heagle et al., 2003; Högy and Fangmeier, 2009) |
|  | ↓ | ^§^Carrot and turnip (Azam et al., 2013), ^‡§^cucumber (Dong et al., 2018; Tang et al., 2018), ^‡^fenugreek and spinach (Jain et al., 2007), ^‡^lettuce (Baslam et al., 2012; Pérez-López et al., 2015a), ^†^potato (Kumari and Agrawal, 2014) and ^§^sweet pepper (Piñero et al., 2017b) |
| Mn | ↑ | ^§^Carrot, radish and turnip (Azam et al., 2013), ^‡^lettuce (Baslam et al., 2012; Pérez-López et al., 2015a; Pérez-López et al., 2015b), ^†^potato (Kumari and Agrawal, 2014) and ^§^sweet pepper (Piñero et al., 2017b) |
|  | = | ^‡^Chinese cabbage (Reich et al., 2016), ^‡§^cucumber (Dong et al., 2018; Tang et al., 2018), ^‡^lettuce (Pérez-López et al., 2015a; Pérez-López et al., 2015b), ^†‡^potato (Fangmeier et al., 2002; Heagle et al., 2003; Högy and Fangmeier, 2009) and ^‡§^sweet pepper (Piñero et al., 2017a; Piñero et al., 2017b) |
|  | ↓ | ^‡^Chinese cabbage (Reich et al., 2016), ^§^cucumber (Dong et al., 2018), ^‡^lettuce (Baslam et al., 2012), ^‡^sweet pepper (Piñero et al., 2017a) and ^§^tomato (Khan et al., 2013) |
| Cu | ↑ | ^§^Carrot and turnip (Azam et al., 2013), ^‡^lettuce (Pérez-López et al., 2015a; Pérez-López et al., 2015b), ^†^potato (Kumari and Agrawal, 2014), ^§^sweet pepper (Piñero et al., 2017b) and ^§^tomato (Khan et al., 2013) |
|  | = | ^‡^Chinese cabbage (Reich et al., 2016), ^‡§^cucumber (Dong et al., 2018), ^‡^lettuce (Baslam et al., 2012; Pérez-López et al., 2015a; Pérez-López et al., 2015b) and ^†‡^potato (Heagle et al., 2003) |
|  | ↓ | ^‡§^Cucumber (Dong et al., 2018), ^‡^lettuce (Baslam et al., 2012), ^§^radish(Azam et al., 2013) and ^§^sweet pepper (Piñero et al., 2017b) |
| Zn | ↑ | ^§^Cucumber (Dong et al., 2018), ^‡^lettuce (Baslam et al., 2012; Pérez-López et al., 2015a) and ^†^potato (Kumari and Agrawal, 2014) |
|  | = | ^‡^Chinese cabbage (Reich et al., 2016), ^‡§^cucumber (Dong et al., 2018; Tang et al., 2018), ^‡^lettuce (Baslam et al., 2012; Pérez-López et al., 2015a; Pérez-López et al., 2015b), ^†‡^potato (Fangmeier et al., 2002; Högy and Fangmeier, 2009) and ^§^sweet pepper (Piñero et al., 2017b) |
|  | ↓ | ^§^Carrot, radish and turnip (Azam et al., 2013), ^‡^Chinese cabbage (Reich et al., 2016), ^‡§^cucumber (Dong et al., 2018; Tang et al., 2018), ^‡^lettuce (Baslam et al., 2012), ^†‡^potato (Heagle et al., 2003; Kumari and Agrawal, 2014) and ^§^tomato (Khan et al., 2013) |
| B | ↑ | ^‡^Lettuce (Pérez-López et al., 2015a; Pérez-López et al., 2015b) |
|  | = | ^‡^Lettuce (Pérez-López et al., 2015a; Pérez-López et al., 2015b), ^†^potato (Högy and Fangmeier, 2009) and ^‡§^sweet pepper (Piñero et al., 2017a; Piñero et al., 2017b) |
|  | ↓ | ^§^Sweet pepper (Piñero et al., 2017b) |
| Mo | = | ^‡^Chinese cabbage (Reich et al., 2016) and ^‡^cucumber (Dong et al., 2018) |
|  | ↓ | ^§^Cucumber (Dong et al., 2018) |
| Na | = | ^‡§^Cucumber (Dong et al., 2018; Tang et al., 2018), ^‡^lettuce (Pérez-López et al., 2015a), ^‡^sweet pepper (Piñero et al., 2017a) and ^†^sugar beet (Manderscheid et al., 2010) |
|  | ↓ | ^§^Cucumber (Tang et al., 2018), ^‡^lettuce (Pérez-López et al., 2015a; Pérez-López et al., 2015b), ^†^potato (Kumari and Agrawal, 2014) and ^‡^sweet pepper (Piñero et al., 2017a) |
| Al | = | ^†^Potato (Högy and Fangmeier, 2009) |
| Cd | = | ^§^Carrot and turnip (Azam et al., 2013) and ^†^potato (Högy and Fangmeier, 2009) |
|  | ↓ | ^§^Tomato (Khan et al., 2013) |
| Cr | = | ^§^Radish and turnip (Azam et al., 2013) |
|  | ↓ | ^§^Carrot (Azam et al., 2013) and ^§^tomato (Khan et al., 2013) |
| Ni | = | ^§^Carrot and radish (Azam et al., 2013) |
|  | ↓ | ^§^Turnip (Azam et al., 2013) and ^§^tomato (Khan et al., 2013) |
| Pb | = | ^§^Carrot, radish and turnip (Azam et al., 2013) |
|  | ↓ | ^§^Tomato (Khan et al., 2013) |

↑ increase, ＝ no significant change, ↓ decrease (*p*<0.05). †, ‡ and § indicate the ratios of concentrations of elevated CO_2_ to ambient CO_2_ are <1.5, 1.5-2.0 and >2.0, respectively.

**Table S6. Effects of elevated CO_2_ on concentrations of other compounds in vegetables.**

| Quality parameters | CO_2_ effects | Crops | |
| --- | --- | --- | --- |
| α-chaconine | = | ^†‡^Potato (Donnelly et al., 2001; Högy and Fangmeier, 2009; Vorne et al., 2002) | |
|  | ↓ | ^†‡^Potato (Högy and Fangmeier, 2009; Vorne et al., 2002) | |
| α-solanine | = | ^†‡^Potato (Donnelly et al., 2001; Högy and Fangmeier, 2009; Vorne et al., 2002) | |
| Aliphatic glucosinolates | ↑ | ^‡^Broccoli (Schonhof et al., 2007) and ^§^Chines kale (La et al., 2009) | |
|  | = | ^‡^Cabbage (Reddy et al., 2004) | |
| Aromatic glucosinolates | = | ^‡^Cabbage (Reddy et al., 2004) | |
| Ash | = | ^§^Carrot, radish and turnip (Azam et al., 2013) and ^§^tomato (Khan et al., 2013) | |
|  | ↓ | ^§^Tomato (Khan et al., 2013) | |
| Capsaicin | ↑/=/↓ | ^†^Hot pepper (Li et al., 2017) | |
| Chloride | = | ^†^Potato (Högy and Fangmeier, 2009) | |
| Cadaverine | ↑/↓ | ^§^Sweet pepper (Piñero et al., 2017b) | |
| Fat | ↓ | ^§^Carrot, radish and turnip (Azam et al., 2013) and ^§^tomato (Khan et al., 2013) |  |
| Glycoalkaloids | ↑ | ^§^Potato (Nitithamyong et al., 1999) | |
|  | = | ^‡^Potato (Donnelly et al., 2001; Vorne et al., 2002) | |
|  | ↓ | ^†‡^Potato (Högy and Fangmeier, 2009; Vorne et al., 2002) | |
| Glycine betaine | ↓/= | ^‡^Sugar beet (Demmers-Derks et al., 1998) | |
| Indolyl glucosinolates | = | ^‡^Cabbage (Reddy et al., 2004) and ^§^Chinese kale (La et al., 2009) | |
|  | ↓ | ^‡^Broccoli (Schonhof et al., 2007) | |
| pH | ↑ | ^‡^Onion (Bettoni et al., 2017) | |
|  | = | ^‡^Onion (Bettoni et al., 2017) and ^§^tomato (Özçelik and Akilli, 1999) | |
| Putrescine | ↑ | ^§^Sweet pepper (Piñero et al., 2017b) | |
| Sulphate | = | ^‡^Chinese cabbage (Reich et al., 2016) and ^†^potato (Högy and Fangmeier, 2009) | |
| Total soluble solids | ↑ | ^‡^Onion (Bettoni et al., 2017) and ^‡§^tomato (Behboudian and Tod, 1995; Helyes et al., 2012; Wei et al., 2018; Zhang et al., 2014) |  |
|  | = | ^‡^Hot pepper (Li et al., 2017), ^‡^onion (Bettoni et al., 2017) and ^†‡§^tomato (Li et al., 1999; Mamatha et al., 2014; Özçelik and Akilli, 1999; Wei et al., 2018; Zhang et al., 2014) |  |
|  | ↓ | ^†‡§^Hot pepper (Li et al., 2017) and ^‡^tomato (Helyes et al., 2012; Mamatha et al., 2014) |  |
| Volatile terpenoids | ↓ | ^§^Hongfengcai (Ren et al., 2014) | |

↑ increase, ＝ no significant change, ↓ decrease (*p*<0.05). †, ‡ and § indicate the ratios of concentrations of elevated CO_2_ to ambient CO_2_ are <1.5, 1.5-2.0 and >2.0, respectively.

**
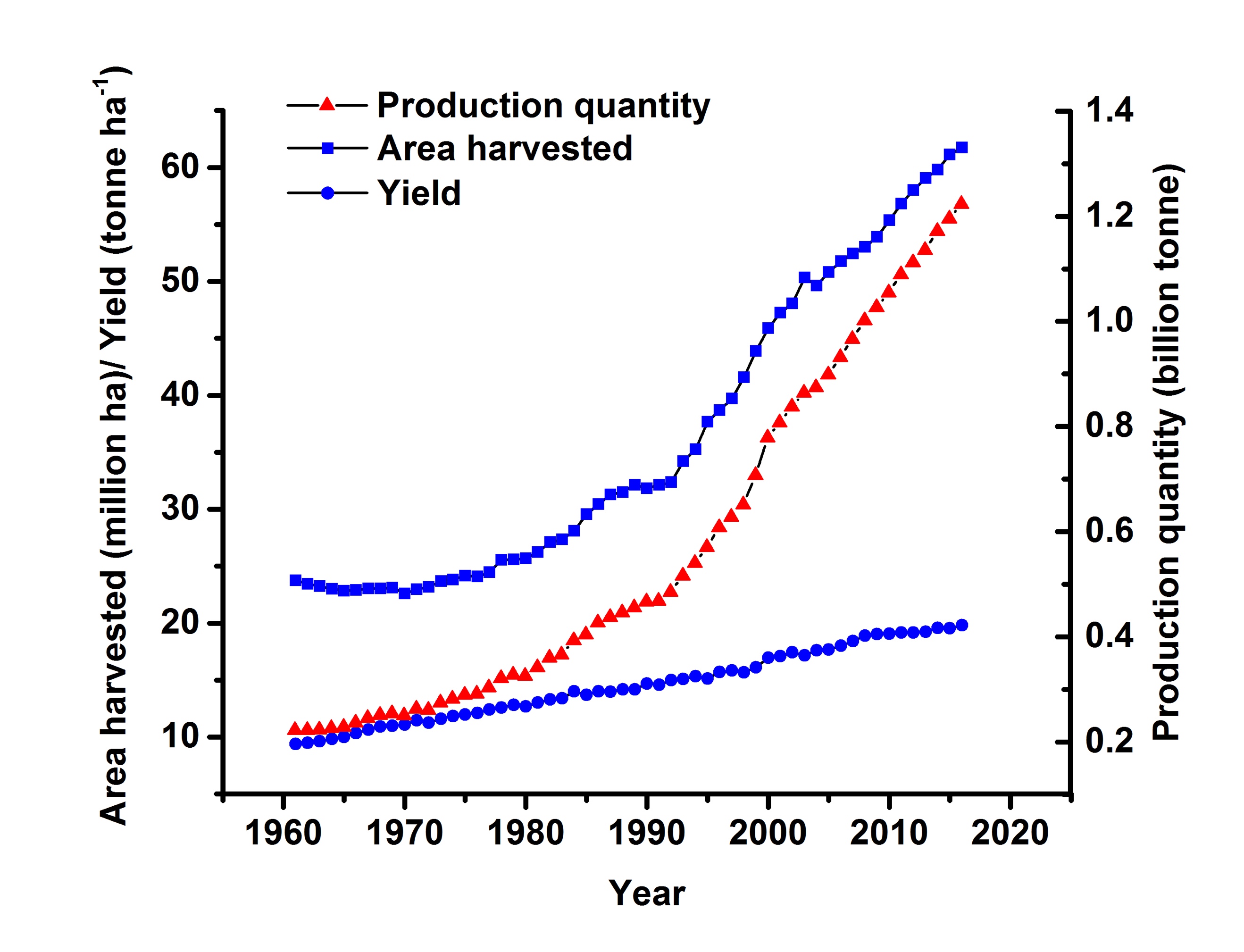
Figure S1. The production, harvested area and yield of total vegetables from 1961 to 2016 in the whole world.** The data were downloaded from the website of FAO STAT (FAOSTAT, 2017). FAOSTAT is founded by the Food and Agriculture Organization of the United Nations, which provides free access to the data related to food and agriculture from over 245 countries or territories since 1961.

**

Figure S2. The relationship of the sample size (number of replicates) with the effect size (nature log of response ratio, %) to test the publication bias.** The dash line and solid line represents the zero line and mean effect size of all parameters used in this study, respectively. “E” and “A” represent for parameters collected under elevated and ambient CO_2_, respectively.

**References:**

Azam, A., Khan, I., Mahmood, A. and Hameed, A. (2013). Yield, chemical composition and nutritional quality responses of carrot, radish and turnip to elevated atmospheric carbon dioxide. J. Sci. Food Agric. , 93, 3237-3244. doi: 10.1002/jsfa.6165

Baslam, M., Garmendia, I. and Goicoechea, N. (2012). Elevated CO_2_ may impair the beneficial effect of arbuscular mycorrhizal fungi on the mineral and phytochemical quality of lettuce. Ann Appl Biol, 161, 180-191. doi: 10.1111/j.1744-7348.2012.00563.x

Becker, C. and Kläring, H.-P. (2016). CO_2_ enrichment can produce high red leaf lettuce yield while increasing most flavonoid glycoside and some caffeic acid derivative concentrations. Food Chem, 199, 736-745. doi: 10.1016/j.foodchem.2015.12.059

Behboudian, M.H. and Tod, C. (1995). Postharvest attributes of `Virosa' tomato fruit produced in an enriched carbon dioxide environment. HortSci, 30, 490-491.

Bettoni, M.M., Mogor, Á.F., Pauletti, V. and Goicoechea, N. (2017). The interaction between mycorrhizal inoculation, humic acids supply and elevated atmospheric CO_2_ increases energetic and antioxidant properties and sweetness of yellow onion. Hortic Environ Biotechnol, 58, 432-440. doi: 10.1007/s13580-017-0122-4

Demmers-Derks, H., Mitchell, R.A.C., Mitchell, V.J. and Lawlor, D.W. (1998). Response of sugar beet (*Beta vulgaris* L.) yield and biochemical composition to elevated CO_2_ and temperature at two nitrogen applications. Plant Cell Environ., 21, 829-836. doi: 10.1046/j.1365-3040.1998.00327.x

Dong, J., Xu, Q., Gruda, N., Chu, W., Li, X. and Duan, Z. (2018). Elevated and super-elevated CO_2_ differ in their interactive effects with nitrogen availability on fruit yield and quality of cucumber. J. Sci. Food Agric. doi: 10.1002/jsfa.8976

Donnelly, A., Lawson, T., Craigon, J., Black, C.R., Colls, J.J. and Landon, G. (2001). Effects of elevated CO_2_ and O_3_ on tuber quality in potato (*Solanum tuberosum* L.). Agric. Ecosyst. Environ., 87, 273-285. doi: 10.1016/S0167-8809(01)00144-X

Fangmeier, A., De Temmerman, L., Black, C., Persson, K. and Vorne, V. (2002). Effects of elevated CO_2_ and/or ozone on nutrient concentrations and nutrient uptake of potatoes. Eur. J. Agron. , 17, 353-368. doi: 10.1016/S1161-0301(02)00071-0

FAOSTAT (2017). Data: Crops. 2017. [http://www.fao.org/faostat/en/ - data/QC](http://www.fao.org/faostat/en/#data/QC)

Fu, Y., Shao, L., Liu, H., Li, H., Zhao, Z., Ye, P., Chen, P. and Liu, H. (2015). Unexpected decrease in yield and antioxidants in vegetable at very high CO_2_ levels. Environ. Chem. Lett., 13, 473-479. doi: 10.1007/s10311-015-0522-6

Ghasemzadeh, A., Jaafar, H.Z.E. and Rahmat, A. (2010). Elevated carbon dioxide increases contents of flavonoids and phenolic compounds, and antioxidant activities in Malaysian young ginger (*Zingiber officinale* Roscoe.) varieties. Molecules, 15, 7907-7922.

Heagle, A.S., Miller, J.E. and Pursley, W.A. (2003). Growth and yield responses of potato to mixtures of carbon dioxide and ozone. J. Environ. Qual., 32, 1603-1610. doi: 10.2134/jeq2003.1603

Helyes, L., Lugasi, A., Neményi, A. and Pék, Z. (2012). The simultaneous effect of elevated CO_2_-level and nitrogen-supply on the fruit components of tomato. Acta Aliment, 41, 265-271. doi: 10.1556/AAlim.41.2012.2.13

Högy, P. and Fangmeier, A. (2009). Atmospheric CO_2_ enrichment affects potatoes: 2. Tuber quality traits. Eur. J. Agron. , 30, 85-94. doi: 10.1016/j.eja.2008.07.006

Islam, M., Matsui, T. and Yoshida, Y. (1994). Effects of carbon dioxide enrichment on acid invertase and sugar concentration in developing tomato fruit. Environ Control Biol, 32, 245-251. doi: 10.2525/ecb1963.32.245

Islam, S., Md, Matsui, T. and Yoshida, Y. (1996). Effect of carbon dioxide enrichment on physico-chemical and enzymatic changes in tomato fruits at various stages of maturity. Sci. Hortic. , 65, 137-149. doi: 10.1016/0304-4238(95)00867-5

Jain, V., Pal, M., Raj, A. and Khetarpal, S. (2007). Photosynthesis and nutrient composition of spinach and fenugreek grown under elevated carbon dioxide concentration. Biol. Plant. , 51, 559-562. doi: 10.1007/s10535-007-0122-9

Jin, C., Du, S., Wang, Y., Condon, J., Lin, X. and Zhang, Y. (2009). Carbon dioxide enrichment by composting in greenhouses and its effect on vegetable production. J. Plant Nutr. Soil Sci., 172, 418-424.

Khan, I., Azam, A. and Mahmood, A. (2013). The impact of enhanced atmospheric carbon dioxide on yield, proximate composition, elemental concentration, fatty acid and vitamin C contents of tomato (*Lycopersicon esculentum*). Environ. Monit. Assess., 185, 205-214. doi: 10.1007/s10661-012-2544-x

Krumbein, A., Kläring, H.-P., Schonhof, I. and Schreiner, M. (2010). Atmospheric carbon dioxide changes photochemical activity, soluble sugars and volatile levels in Broccoli (*Brassica oleracea* var. italica). J. Agric. Food Chem., 58, 3747-3752. doi: 10.1021/jf903280w

Krumbein, A., Schwarz, D. and Kläring, H.P. (2006). Effects of environmental factors on carotenoid content in tomato (*Lycopersicon esculentum* L. Mill.) grown in a greenhouse. J. Appl. Bot. Food Qual., 80, 160-164.

Kumari, S. and Agrawal, M. (2014). Growth, yield and quality attributes of a tropical potato variety (*Solanum tuberosum* L. cv Kufri chandramukhi) under ambient and elevated carbon dioxide and ozone and their interactions. Ecotoxicol Environ Saf., 101, 146-156. doi: 10.1016/j.ecoenv.2013.12.021

Kumari, S., Agrawal, M. and Tiwari, S. (2013). Impact of elevated CO_2_ and elevated O_3_ on *Beta vulgaris* L.: Pigments, metabolites, antioxidants, growth and yield. Environ Pollut., 174, 279-288. doi: 10.1016/j.envpol.2012.11.021

La, G., Fang, P., Teng, Y., Li, Y. and Lin, X. (2009). Effect of CO_2_ enrichment on the glucosinolate contents under different nitrogen levels in bolting stem of Chinese kale (*Brassica alboglabra* L.). J Zhejiang Univ Sci B, 10, 454-464. doi: 10.1631/jzus.B0820354

Levine, L.H. and Paré, P.W. (2009). Antioxidant capacity reduced in scallions grown under elevated CO_2_ independent of assayed light intensity. Adv. Space Res., 44, 887-894. doi: 10.1016/j.asr.2009.06.017

Li, F., Wang, J., Chen, Y., Zou, Z., Wang, X. and Yue, M. (2007). Combined effects of enhanced ultraviolet-B radiation and doubled CO_2_ concentration on growth, fruit quality and yield of tomato in winter plastic greenhouse. Front. Biol. China 2, 414-418. doi: 10.1007/s11515-007-0063-x

Li, J.H., Sagi, M., Gale, J., Volokita, M. and Novoplansky, A. (1999). Response of tomato plants to saline water as affected by carbon dioxide supplementation. I. Growth, yield and fruit quality. J. Hortic. Sci. Biotechnol., 74, 232-237. doi: 10.1080/14620316.1999.11511100

Li, X., Kang, S., Li, F., Zhang, X., Huo, Z., Ding, R., Tong, L., Du, T. and Li, S. (2017). Light supplement and carbon dioxide enrichment affect yield and quality of off-season pepper. Agron. J., 109, 2107-2118. doi: 10.2134/agronj2017.01.0044

Liu, J., Feng, K., Wang, G., Xu, Z., Wang, F. and Xiong, A. (2018). Elevated CO_2_ induces alteration in lignin accumulation in celery (*Apium graveolens* L.). Plant Physiol Biochem, 127, 310-319. doi: 10.1016/j.plaphy.2018.04.003

Mamatha, H., Srinivasa Rao, N.K., Laxman, R.H., Shivashankara, K.S., Bhatt, R.M. and Pavithra, K.C. (2014). Impact of elevated CO_2_ on growth, physiology, yield, and quality of tomato (*Lycopersicon esculentum* Mill) cv. Arka Ashish. Photosynthetica, 52, 519-528. doi: 10.1007/s11099-014-0059-0

Manderscheid, R., Pacholski, A. and Weigel, H.-J. (2010). Effect of free air carbon dioxide enrichment combined with two nitrogen levels on growth, yield and yield quality of sugar beet: Evidence for a sink limitation of beet growth under elevated CO_2_. Eur. J. Agron. , 32, 228-239. doi: 10.1016/j.eja.2009.12.002

Miyagi, A., Uchimiya, H. and Kawai-Yamada, M. (2017). Synergistic effects of light quality, carbon dioxide and nutrients on metabolite compositions of head lettuce under artificial growth conditions mimicking a plant factory. Food Chem., 218, 561-568. doi: 10.1016/j.foodchem.2016.09.102

Nitithamyong, A., Vonelbe, J.H., Wheeler, R.M. and Tibbitts, T.W. (1999). Glycoalkaloids in potato tubers grown under controlled environments. Am J Potato Res, 76, 337-343. doi: 10.1007/BF02910006

Özçelik, N. and Akilli, M. (1999). Effects of CO_2_ enrichment on vegetative growth, yield and quality of greenhouse-grown tomatoes in soil and soilless cultures. ActaHortic., 486, 155-160. doi: 10.17660/ActaHortic.1999.491.22

Pérez-López, U., Miranda-Apodaca, J., Lacuesta, M., Mena-Petite, A. and Muñoz-Rueda, A. (2015a). Growth and nutritional quality improvement in two differently pigmented lettuce cultivars grown under elevated CO_2_ and/or salinity. Sci. Hortic., 195, 56-66. doi: 10.1016/j.scienta.2015.08.034

Pérez-López, U., Miranda-Apodaca, J., Muñoz-Rueda, A. and Mena-Petite, A. (2013). Lettuce production and antioxidant capacity are differentially modified by salt stress and light intensity under ambient and elevated CO_2_. J. Plant Physiol., 170, 1517-1525. doi: 10.1016/j.jplph.2013.06.004

Pérez-López, U., Miranda-Apodaca, J., Muñoz-Rueda, A. and Mena-Petite, A. (2015b). Interacting effects of high light and elevated CO_2_ on the nutraceutical quality of two differently pigmented *Lactuca sativa* cultivars (Blonde of Paris Batavia and Oak Leaf). Sci. Hortic. , 191, 38-48. doi: 10.1016/j.scienta.2015.04.030

Pérez-López, U., Sgherri, C., Miranda-Apodaca, J., Micaelli, F., Lacuesta, M., Mena-Petite, A., Quartacci, M.F. and Muñoz-Rueda, A. (2018). Concentration of phenolic compounds is increased in lettuce grown under high light intensity and elevated CO_2_. Plant Physiol Biochem, 123, 233-241. doi: 10.1016/j.plaphy.2017.12.010

Piñero, M., Carmen, Pérez‐Jiménez, M., López‐Marín, J. and del Amor Francisco, M. (2017a). Fruit quality of sweet pepper as affected by foliar Ca applications to mitigate the supply of saline water under a climate change scenario. J. Sci. Food Agric. , 98, 1071-1078. doi: 10.1002/jsfa.8557

Piñero, M.C., Otálora, G., Porras, M.E., Sánchez-Guerrero, M.C., Lorenzo, P., Medrano, E. and del Amor, F.M. (2017b). The form in which nitrogen is supplied affects the polyamines, amino acids, and mineral composition of sweet pepper fruit under an elevated CO_2_ concentration. J. Agric. Food Chem., 65, 711-717. doi: 10.1021/acs.jafc.6b04118

Proietti, S., Moscatello, S., Giacomelli, G.A. and Battistelli, A. (2013). Influence of the interaction between light intensity and CO_2_ concentration on productivity and quality of spinach (*Spinacia oleracea* L.) grown in fully controlled environment. Adv. Space Res., 52, 1193-1200. doi: 10.1016/j.asr.2013.06.005

Reddy, G.V.P., Tossavainen, P., Nerg, A.-M. and Holopainen, J.K. (2004). Elevated atmospheric CO_2_ affects the chemical quality of brassica plants and the growth rate of the specialist, *Plutella xylostella*, but not the generalist, *Spodoptera littoralis*. J. Agric. Food Chem., 52, 4185-4191. doi: 10.1021/jf049358v

Reich, M., van den Meerakker, A.N., Parmar, S., Hawkesford, M.J. and De Kok, L.J. (2016). Temperature determines size and direction of effects of elevated CO_2_ and nitrogen form on yield quantity and quality of Chinese cabbage. Plant Biol, 18, 63-75. doi: 10.1111/plb.12396

Ren, J., Guo, S.-S., Xin, X.-L. and Chen, L. (2014). Changes in volatile constituents and phenols from *Gynura bicolor* DC grown in elevated CO_2_ and LED lighting. Sci. Hortic. , 175, 243-250. doi: 10.1016/j.scienta.2014.06.023

Schonhof, I., Kläring, H.P., Krumbein, A. and Schreiner, M. (2007). Interaction between atmospheric CO_2_ and glucosinolates in Broccoli. J. Chem. Ecol, 33, 105-114. doi: 10.1007/s10886-006-9202-0

Seo, Y., Ide, K., Kitahata, N., Kuchitsu, K. and Dowaki, K. (2017). Environmental impact and nutritional improvement of elevated CO_2_ treatment: a case study of spinach production. Sustainability, 9. doi: 10.3390/su9101854

Sgherri, C., Pérez-López, U., Micaelli, F., Miranda-Apodaca, J., Mena-Petite, A., Muñoz-Rueda, A. and Quartacci, M.F. (2017). Elevated CO_2_ and salinity are responsible for phenolics-enrichment in two differently pigmented lettuces. Plant Physiol Biochem, 115, 269-278. doi: 10.1016/j.plaphy.2017.04.006

Sun, P., Mantri, N., Lou, H., Hu, Y., Sun, D., Zhu, Y., Dong, T. and Lu, H. (2012). Effects of elevated CO_2_ and temperature on yield and fruit quality of strawberry (*Fragaria × ananassa* Duch.) at two levels of nitrogen application. Plos One, 7, e41000. doi: 10.1371/journal.pone.0041000

Tang, Y., Dong, J., Li, X., Gruda, N. and Duan, Z. (2018). Interactive effects of elevated carbon dioxide and nitrogen availability on fruit quality of cucumber (*Cucumis sativus* L.). J Integr Agric., In press.

Thompson, L., Peffley, E., Green, C., Pare, P. and Tissue, D., 2004. Biomass, flavonol levels and sensory characteristics of Allium cultivars grown hydroponically at ambient and elevated CO_2_, Proceedings of the 34th International Conferences on Environmental Systems (ICES), Colorado Springs, Colorado.

Vorne, V., Ojanperä, K., De Temmerman, L., Bindi, M., Högy, P., Jones, M., Lawson, T. and Persson, K. (2002). Effects of elevated carbon dioxide and ozone on potato tuber quality in the European multiple-site experiment ‘CHIP-project’. Eur. J. Agron. , 17, 369-381.

Wang, M., Liu, H., Dong, C., Fu, Y. and Liu, H. (2016). Elevated CO_2_ enhances photosynthetic efficiency, ion uptake and antioxidant activity of *Gynura bicolor* DC. grown in a porous-tube nutrient delivery system under simulated microgravity. Plant Biol, 18, 391-399. doi: 10.1111/plb.12426

Wang, S.Y. and Bunce, J.A. (2004). Elevated carbon dioxide affects fruit flavor in field-grown strawberries (*Fragaria × ananassa* Duch). J. Sci. Food Agric., 84, 1464-1468. doi: 10.1002/jsfa.1824

Wang, S.Y., Bunce, J.A. and Maas, J.L. (2003). Elevated carbon dioxide increases contents of antioxidant compounds in field-grown strawberries. J. Agric. Food Chem., 51, 4315-4320. doi: 10.1021/jf021172d

Wei, Z., Du, T., Li, X., Fang, L. and Liu, F. (2018). Interactive effects of elevated CO_2_ and N fertilization on yield and quality of tomato grown under reduced irrigation regime. Front. Plant Sci., 9. doi: 10.3389/fpls.2018.00328

Wheeler, R.M., Mackowiak, C.L., Stutte, G.W., Yorio, N.C. and Berry, W.L. (1997). Effect of elevated carbon dioxide on nutritional quality of tomato. Adv. Space Res., 20, 1975-1978. doi: 10.1016/S0273-1177(97)00263-9

Zhang, Z., Liu, L., Zhang, M., Zhang, Y. and Wang, Q. (2014). Effect of carbon dioxide enrichment on health-promoting compounds and organoleptic properties of tomato fruits grown in greenhouse. Food Chem., 153, 157-163. doi: 10.1016/j.foodchem.2013.12.052
